# R/G Value—A Numeric Index of Individual Periodontal Health and Oral Microbiome Dynamics

**DOI:** 10.3389/fcimb.2021.602643

**Published:** 2021-03-10

**Authors:** Lucie Najmanova, Lenka Sabova, Magdalena Lenartova, Tatjana Janatova, Jaroslav Mysak, Tomas Vetrovsky, Barbora Tesinska, Gabriela Balikova Novotna, Marketa Koberska, Zdenek Broukal, Jana Duskova, Stepan Podzimek, Jiri Janata

**Affiliations:** ^1^ Institute of Microbiology v. v. i., Czech Academy of Sciences, Prague, Czechia; ^2^ Department of Genetics and Microbiology, Faculty of Science, Charles University, Prague, Czechia; ^3^ Institute of Dental Medicine, First Faculty of Medicine, Charles University and General University Hospital in Prague, Prague, Czechia; ^4^ Institute of Microbiology v. v. i., BIOCEV, Czech Academy of Sciences, Vestec, Czechia

**Keywords:** oral microbiome, periodontal health, periodontitis, core microbiome, temporal dynamics, taxonomic composition, diagnosis

## Abstract

The dysbiosis of oral microbiome (OM) precedes the clinical signs of periodontal disease. Its simple measure thus could indicate individuals at risk of periodontitis development; however, such a tool is still missing. Up to now, numerous microbial taxa were associated with periodontal health or periodontitis. The outputs of most studies could, nevertheless, be slightly biased from following two reasons: First, the healthy group is often characterized only by the absence of the disease, but the individuals could already suffer from dysbiosis without any visible signs. Second, the healthy/diseased OM characteristics are frequently determined based on average data obtained for whole groups of periodontally healthy persons *versus* patients. Especially in smaller sets of tested individuals the typical individual variability can thus complicate the unambiguous assignment of oral taxa to respective state of health. In this work the taxonomic composition of OM was evaluated for 20 periodontally healthy individuals and 15 patients with chronic periodontitis. The narrowed selection set of the most diseased patients (confirmed by clinical parameters) and the most distant group of healthy individuals with the lowest probability of dysbiosis was determined by clustering analysis and used for identification of marker taxa. Based on their representation in each individual oral cavity we proposed the numeric index of periodontal health called R/G value. Its diagnostic potential was further confirmed using independent set of 20 periodontally healthy individuals and 20 patients with periodontitis with 95 percent of samples assigned correctly. We also assessed the individual temporal OM dynamics in periodontal health and we compared it to periodontitis. We revealed that the taxonomic composition of the system changes dynamically but generally it ranges within values typical for periodontal health or transient state, but far from values typical for periodontitis. R/G value tool, formulated from individually evaluated data, allowed us to arrange individual OMs into a continuous series, instead of two distinct groups, thus mimicking the gradual transformation of a virtual person from periodontal health to disease. The application of R/G value index thus represents a very promising diagnostic tool for early prediction of persons at risk of developing periodontal disease.

## Introduction

Periodontitis is a multifactorial disease of the tooth-supporting tissues where the genetic predisposition and physiological state of the host take place, however, the active role of subgingival bacterial biofilm in tissue damage is known to be the crucial factor in the pathogenesis. Periodontitis has a polymicrobial etiology within a framework of the complex oral microbiome (OM). Decades of investigations have demonstrated that the microbial communities associated with periodontitis differ from those in healthy individuals (Socransky and Haffajee, 2005; Abusleme et al., 2013; Pérez-Chaparro et al., 2014; Boutin et al., 2017). In the last several years, dozens of studies were published employing next-generation sequencing methods for the general characterization of the oral cavity microbiome. Many of them (Griffen et al., 2012; Abusleme et al., 2013; Kistler et al., 2013; Wang et al., 2013; Camelo-Castillo et al., 2015; Kirst et al., 2015; Szafranski et al., 2015) compare the OM of patients with periodontitis to that of healthy individuals. All these studies provide a comprehensive view of the ecological shifts associated with the disease and document higher diversity of the microbial community in periodontitis (Griffen et al., 2012; Li et al., 2014; Shi et al., 2015; Tsai et al., 2018). Griffen (Griffen et al., 2012), for example, identified 123 species to be significantly more abundant in the disease, mainly members of phyla *Spirochaetes*, *Synergistetes* (now *Fretibacteria*), and *Bacteroidetes*, but only 53 in health, most of all *Proteobacteria*. Abusleme (Abusleme et al., 2013) additionally suggested that OM in periodontitis results from microbial succession characterized by the emergence of newly abundant taxa, however without replacement of the health-associated ones. She has defined the core subgingival microbiome, a set of taxa with equal prevalence and relative abundance in health and periodontitis, comprising mainly *Fusobacterium nucleatum* species and with lower abundance but a wide prevalence also *Lautropia mirabilis*, *Corynebacterium matruchotii*, *Campylobacter gracilis*, *Eikenella corrodens*, *Veillonella parvula*, unclassified *Pasteurellaceae*, *Pseudomonas* sp., some *Streptococcus* sp., and *Granulicatella adjacens.* In addition to this core microbiome, the high prevalence and relative abundance of health-associated taxa (*Rothia*, *Actinomyces*, *Streptococcus*, etc.) were detected in healthy controls while *Fretibacteria* and genera *Treponema*, *Porphyromonas*, and *Tannerella* prevailed in periodontitis.

The potential of OM taxonomic composition assessment for early diagnosis of periodontitis is undisputed. Before its employment in clinical practice, however, it is necessary to associate specific taxa unequivocally to periodontal health or periodontitis and to define their mutual relations (co-occurrence networks) to distinguish between physiological dynamics of the system and the genuine dysbiosis leading to the onset of the disease. In yet published studies, the OM composition in health and disease is mostly compared based on average relative abundance values of specific taxa at the group level; i.e. an average value for a group of healthy individuals compared to the average value for diseased persons, alternatively average value before/after treatment, etc.

In this work, we assess the individual OM taxonomic composition dynamics in periodontally healthy persons in three time points (initial sampling at time T0; T1 = T0 plus 2 weeks; and T2 = T1 plus 2 months) and we compare this healthy OM to that of patients with chronic periodontitis. We also employ the procedure described previously (Abusleme et al., 2013) to define health and periodontitis associated oral taxa and further we propose the numeric index to describe the periodontal health status of each individual. We extend the conclusions of the above-cited study of Abusleme to show that the described core microbiome rather represents the transition state on the way from periodontal health to periodontitis and in pronounced cases of completely healthy or severely diseased persons it almost disappears to favor health or periodontitis associated taxa.

## Materials and Methods

### Participants (Clinical Characteristics of Tested Subjects)

In the manuscript, we employ following sets of subjects:

The full basic set comprising 15 adult patients with chronic periodontitis (P1–15) and 20 periodontally healthy subjects (H1–20), from which a selection narrowed subset of 9 most diseased patients (sP) and opposite 10 healthy (sH) individuals were defined based on NMDS (see *Results: The overall OM composition of H differs from that of P* and **Supplementary Table 1**). To be included in the H group the subjects were required to have no periodontal pockets on probing depth >3 mm. In the P group, each subject had minimally 16 natural teeth and at least 2 periodontal pockets on probing depth >6 mm. The number of periodontal pockets in P varied from 2 to 20. Both groups were gender-balanced. The average age was 49 years for P (42–66) and 41 years for H (25–68). The subjects had not received any antibiotic treatment or periodontal therapy in the last 3 months before the beginning of the study and besides periodontitis (in P group) they were in good general health. For additional clinical characteristics (number of affected teeth, PPD, BOP, CAL) and also calculated R/G value of all subjects of the full basic set see **Supplementary Table 1**.

An independent testing set of another 20 periodontally healthy (TH1–20) and 20 diseased (TP1–20) individuals was employed to confirm the diagnostic potential of the proposed R/G value. For the detailed clinical characteristics (number of affected teeth, PPD, BOP, CAL) and also calculated R/G value of the set see **Supplementary Table 5**, list 1 “Testing set all proband charact”.

The study has been approved by the Ethics Committee of the First Faculty of Medicine of Charles University and General University Hospital in Prague as a part of project No. 17-30753A of the Czech Health Research Council and accordingly all human subjects involved in the study signed the informed written consent.

### The Study Design and Sample Collection

To reveal the natural individual dynamics of OM composition, the H subjects were sampled three times with interval 14 days (time T1) from the initial sampling (time T0) and plus another 2 months (time T2). The sampling of P subjects occurred prior start of their therapy, i.e. the OM of patients at T0 was not affected by any antibacterial compound, SRP therapy or their combination. The sampling procedure was the following: The gingival crevice fluid was collected using paper points (BECHT, Germany). In patients, two sterile paper points were inserted simultaneously into the depth of the periodontal pocket, and they were left 10 s under the gingiva to soak the fluid, in the H group the samples were taken similarly, but from the gingival buccal sulcus. Both paper points from one sampling were further processed together. The DNA was extracted from paper points using DNeasy Blood&Tissue kit (Qiagen, Germany) according to the manufacturer’s instructions. The DNA precipitate was diluted in 140 µl AE buffer and stored at −20°C for further analysis.

The testing set of 20 TH and 20 TP samples was taken and processed analogously.

### Tag-Encoded Amplicon Pyrosequencing

The procedure of sample preparation and pyrosequencing was adjusted according to Baldrian (Baldrian et al., 2012). The eubacterial primers eub530f/eub1101r (Dowd et al., 2008) were used to amplify the V4–V6 region of bacterial 16S rDNA. Phusion primers for tag-encoded 454-Titanium pyrosequencing contained additionally Titanium A or B adaptors including four nucleotides long key sequence (Roche, Basel, Switzerland) and sample tags, separated from primers by three nucleotide long spacers (Parameswaran et al., 2007). The sequences of all composite primers used in this study are listed in **Supplementary Table 2**.

PCR amplifications were performed in two steps. The robustness and reproducibility of the method were increased by performing the primary PCR (the first step) in three independent 50 µl reactions per sample, which were further pooled and processed together. Each 50 µl reaction contained 5 µl of 10x NzyTaq polymerase buffer, 2 µl of 50 mM MgCl_2_, 2 µl of 10 mM dNTP, 2 µl of each primer (0.25 mM), 34 µl of dH_2_O, 1 µl of 5U/µl Supreme NzyTaq polymerase (NZYTech, Portugal) containing 4% Phusion High-Fidelity DNA polymerase (NEB, USA), and 2 µl of template DNA. The cycling conditions were 94°C for 5 min; 35 cycles of 94°C for 1 min, 60°C for 50 s, and 72°C for 30 s; followed by 72°C for 10 min. Pooled PCR products of the three independent reactions for each sample were purified using the Wizard SV Gel and PCR Clean-Up System (Promega, USA). Then 100 ng of each pooled purified primary amplicon was used as a template in the secondary PCR performed under the same conditions except that phusion primers were used and the number of cycles was 10. PCR products were separated by electrophoresis and gel purified using the Wizard SV Gel and PCR Clean-Up System. The DNA was quantified by qPCR using KAPA Library Quantification Kit (Kapa Biosystems, USA). An equimolar mix of secondary PCR products from all samples was subjected to sequencing on a GS FLX Titanium platform (Roche) using unidirectional amplicon library sequencing protocol. To assess the reproducibility of the method, we performed the whole procedure starting with primary PCR twice independently for all initial samples (time T0), and compared the results. For further analysis, the sequences obtained from parallels for each sample were merged. The raw sequences are deposited in the NCBI Short Read Archive (BioProject accession no. PRJNA291567).

### Analysis of Sequencing Data

The output data were processed using Mothur (Schloss et al., 2009) and sequences shorter than 365 nt, those with average sequence quality lower than 20 or those with ambiguous base calls (N) were filtered out. Subsequently, the barcodes and primer sequences were trimmed off.

The sequences were further processed using SEED 2.0 (Větrovský et al., 2018). The sequences were clustered at 98.5% identity (Uparse, OTU radius 1.5) and the identified chimeric sequences were removed. The consensus sequences for each cluster were generated and compared to HOMD 16S RefSeq 14.51 database (start position 9) using BLAST online tool to determine the matching oral taxa (OT). Many consensus sequences were assigned to the HOMD OT unambiguously but many others could be assigned to more OTs with the same probability. This is because not the whole length, but only a 365 nt long portion of the 16S rDNA sequence was analyzed. To overcome this ambiguous assignment we instead defined the “combined taxa” (CTs). Each CT associates OTs that cannot be distinguished when comparing our 365 nt long portions of 16S rDNA at 98.5% identity. To create CT table, we exported the HOMD 16S RefSeq 14.51 database (start position 9), opened in SEED 2.0, and aligned using MAFFT tool. All sequences were further trimmed analogously to the sequences of our testing dataset, i.e. the forward primer eub530f sequence including all upstream nucleotides were cut off, and further, the sequences were trimmed to 365 nt. The sequences were then clustered (Uparse, OTU radius 1.5) resulting in 106 CTs (containing from 2 to 22 OTs) and 290 OTs which could be assigned unambiguously. These clusters were named CT001–CT106 (for the list of OTs forming each CT see **Supplementary Table 3**).

For each sample, the numbers of sequences assigned to individual OTs/CTs have been determined. However, to estimate the relative abundances of respective taxa (i.e. reflecting their different number of 16S rDNA copies), the values were further normalized to the number of 16S rRNA gene copies per genome, which is taxon-specific and can vary from 1 up to 15 or more (Klappenbach et al., 2001). The detected number of sequences for each taxon has thus been recalculated to respective abundance according to Větrovský and Baldrian (2013) using for each HOMD taxon the 16S rRNA gene copy-number value of the respective phylogenetically closest bacteria with the already sequenced genome. The published 16S rRNA gene copy-number table (Větrovský and Baldrian, 2013) was updated for this purpose according to the database https://rrndb.umms.med.umich.edu/ (Stoddard et al., 2015; **Supplementary Table 4**). The obtained values were expressed as a percentage of the total normalized number of 16S rDNA copies identified in the sample (i.e. as relative abundances). For further analyses only the major taxa (in respect to both the prevalence and relative abundance) were taken into account. The criteria of major taxa selection are specified for each purpose in respective chapter of *Results*.

The following pipeline was employed to evaluate the sequencing results:

1. The OM taxonomic composition was defined for all individual samples.
2. A full set (20 H + 15 P) of initial (T0) samples was analyzed using NMDS and a selection set of 9 most severe patients (sP) and opposite 10 healthy subjects (sH) with the most distinct OM taxonomic composition was defined.
3. The identified major oral taxa, matching the criteria of selection, were assigned to periodontal health (green) or periodontitis (red) based on their relative abundance and prevalence within the selection set of individuals (9 sP + 10 sH).
4. The numeric coefficient of periodontal disease (R/G value) was defined based on the presence of R and G taxa.
5. The R/G value predictive ability was verified on an independent testing set of 20 healthy (TH1–TH20, test healthy) and 20 diseased (TP1–TP20, test periodontitis) probands.

The dynamics of individual OM taxonomic composition in time (T0, T1, and T2) was evaluated for each H person from initial full set (20 H) and it was compared to the OM in periodontitis.

### Calculations and Statistical Analysis

For computational analysis a free software PAST version 3.25 (Hammer et al., 2001) was used: The assortment according to the relative abundance of significant (see above) OTs/CTs was performed using non-metric multidimensional scaling (NMDS, similarity index Bray-Curtis).

To evaluate the reproducibility of the method (comparison of T0 parallels) as well as the OM variability in time, we employed the Bray-Curtis similarity index estimation in PAST 3.25.

Heatmaps were constructed in program R (http://www.r-project.org/) using “heatmap.2” function.

## Results

### The Overall OM Composition of H Differs From That of P

**Figure 1:**
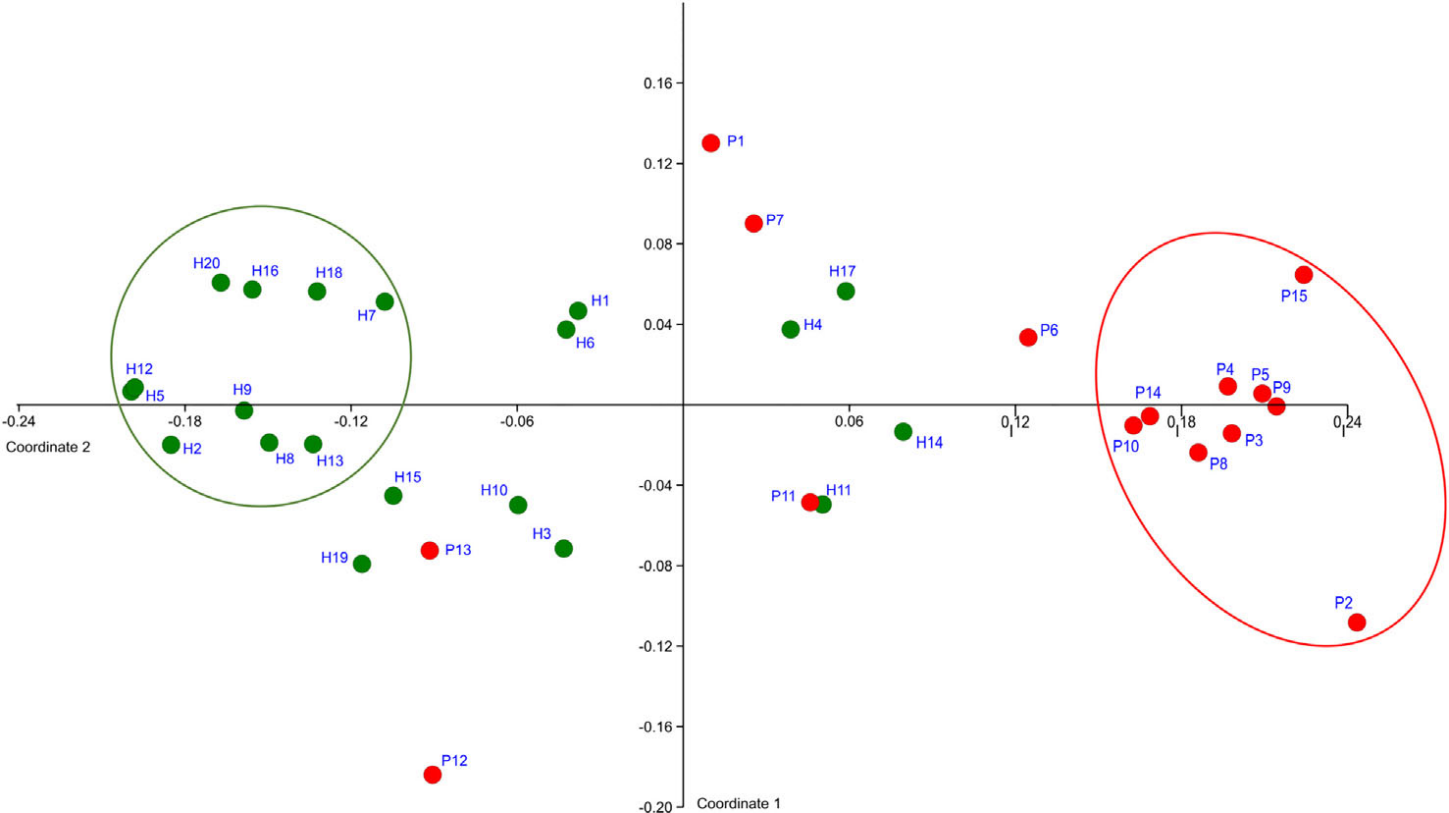
The definition of representative selection sub-sets sP and sH. NMDS analysis plot shows the clustering of subjects with periodontitis (red dots) apart from healthy subjects (green dots). The ovals mark the subjects with obviously distinct bacterial profiles for both groups (representative selection sub-sets).

The OM composition of a full set of 20 H was compared to that of a full set of 15 P. The T0 samples were compared. In total 128,654 sequences were obtained after filtration and the number of sequences per sample ranged from 1,576 to 5,780 for P and from 1,126 to 10,056 for H. The sequences were clustered and assigned to 301 OTs and 102 CTs. Only 58 taxa (OTs or CTs) matched the selection criteria, which we defined as minimal average abundance 0.3% in any of P or H group and minimum prevalence in at least 7 out of 15 P or 10 out of 20 H. Just these major taxa, which, however, represent 87% of normalized reads, were included in further analysis. The NMDS plot created based on the comparison of relative abundances of major taxa, distinguished the samples according to the health status of relevant subjects (**Figure 1**).

A clearly separated compact cluster in red oval (**Figure 1**) corresponds to OM profiles of nine patients (P2, P3, P4, P5, P8, P9, P10, P14, P15), eight of them exhibiting the most severe clinical signs of periodontitis, i.e., >5 affected teeth with periodontal pockets >6 mm (**Supplementary Table 1**). These individuals were selected for a narrowed sP sub-set. A sub-set sH of 10 healthy subjects (H2, H5, H7, H8, H9, H12, H13, H16, H18, H20) was selected from the opposite part of the plot (green circle, **Figure 1**).

### Core Microbiome Represents Rather the Transition State From Health to Periodontitis

**Figure 2:**
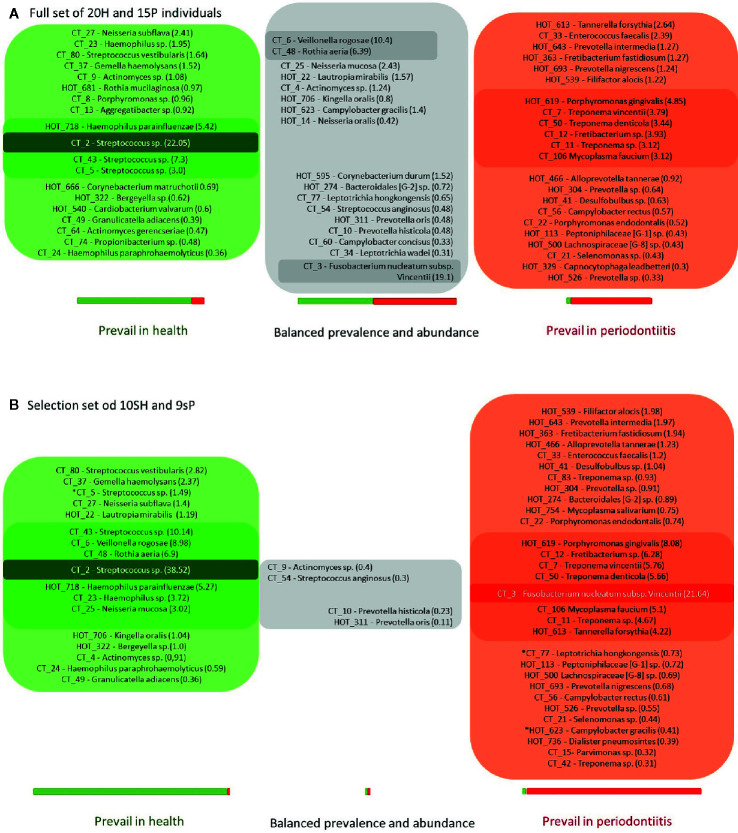
The OM composition in health and periodontitis as defined for full sets (20H + 15P; **A**) or narrowed selection sets (10sH + 9sP; **B**) of tested individuals. The green box: taxa exhibiting the H:P ratio >3; the red box: taxa exhibiting the H:P ratio <0.33; the gray box: remaining taxa exhibiting a balanced distribution in both tested groups. The numbers in parentheses correspond to the average relative abundances in H for taxa with H:P ratio >1 (the green box and the upper part of the gray box) and in P for taxa with H:P ratio <1 (the lower part of the gray box and the red box) with the intensity of the box color referring to this value. The lightest color stands for relative abundance values <3%; the medium for values in the interval from 3 to 20%; the darkest one for values >20%. The length of the green-red bar below the appropriate box corresponds to the total sum of average relative abundances of all taxa assigned to the box in the whole set of tested individuals. The contribution of H (green part) or P (red part) group of subjects is shown in colors. The stars stand for R or G taxa at 2B, which do not meet the criterion H:P ratio >10 or <0.1.

The 58 major taxa (OTs/CTs) identified in the full basic set of 20H + 15P T0 samples were divided into groups according to the ratio of their average overall abundance in H and P (the H:P ratio; **Figure 2A**).

Similarly to Abusleme et al. (2013), who defined a group of core subgingival taxa (taxa, that were identified with equal prevalence and relative abundance in both, periodontal patients and healthy subjects), also in our full basic set of 20H + 15P such a seeming core OM was identified comprising taxa with balanced prevalence in both H and P groups and the H:P ratio ranging from 0.3 to 3 (marked gray in **Figure 2A**). The taxa highly prevalent and abundant in H (exhibiting H:P ratio >3) were marked green and those exhibiting H:P ratio <0.33 were marked red (**Figure 2A**).

A profoundly different picture was obtained, when the equal analysis was performed for narrowed selection sub-set of 10sH + 9sP individuals with the most pronounced state of oral health. The taxa detected in at least four out of nine sP or five out of 10 sH and with average relative abundance minimally 0.3% in at least one group, were considered the major. From 58 major taxa detected for the full basic set of tested individuals, 12 OTs/CTs disappeared (11 of them from green or gray group at **Figure 2A**), and five new major taxa (all assigned to periodontitis, i.e., red group) emerged (**Figure 2B**). In the narrowed selection sub-set, thus only 51 OTs/CTs matched the criteria of selection. At the **Figure 2B**, we can see that among the most diseased and the most healthy individuals, the major taxa formed two distinct groups (green and red) with only a minimal overlap, i.e., the taxa defined by overall comparison of full basic set (15P + 20H) as gray core microbiome, didn’t match the criteria of selection (i.e. were omitted from the analysis), or were clearly assigned or to the green or to the red group. Only four taxa, all exhibiting very low average relative abundance (CTs assigned to *Actinomyces* sp., *Streptococcus angiosus*, *Prevotella histicola*, and OT *Prevotella oris*) remained gray, nevertheless, they form rather a negligible portion of OM of tested individuals in the narrowed sub-set. Moreover, the distribution of the majority of R and G taxa (except for three, marked with stars at **Figure 2B**), met even a stricter rule: the H:P ratio >10 for green taxa and <0.1 for red taxa. In the selection sub-set, the majority of green (health-associated) taxa thus exhibits 10 times higher average relative abundance among sH when compared to sP, and the red (periodontitis associated) taxa exhibit 10 times higher average relative abundance among sP when compared to sH. The assignment of identified taxa as markers of periodontitis (30 taxa in the red box, R) and periodontal health (17 taxa in the green box, G), as shown in **Figure 2B**, was employed in further analysis.

**Figure 3:**
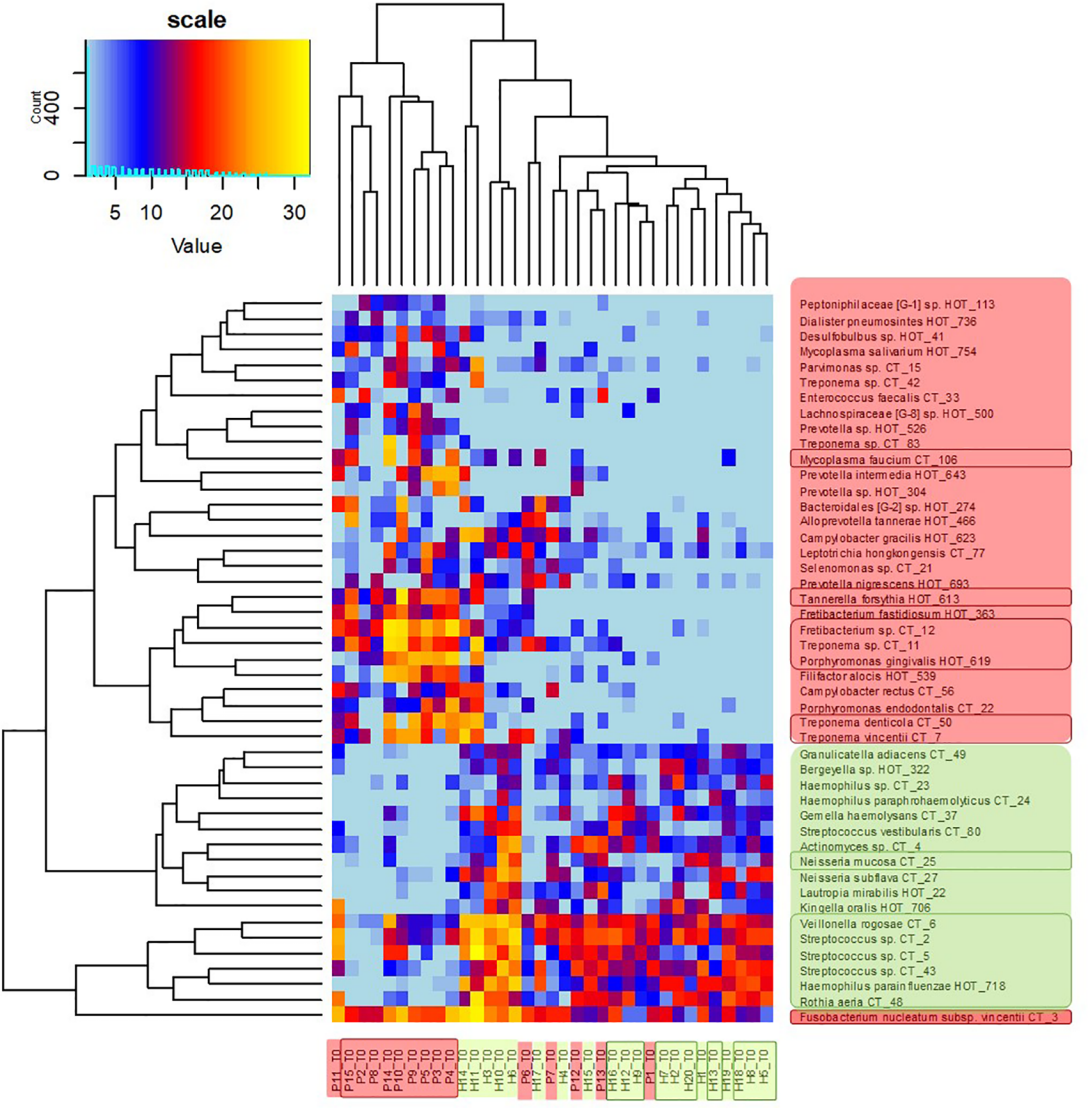
OM profiles of a full set of 20H and 15P at T0 based on 30R and 17G taxa relative abundance. Patients are highlighted in red, healthy individuals in green, members of sP, and sH groups are shown in frames. The taxa names are also color-coded: green for periodontal health and red for periodontitis. Highly abundant taxa (average relative abundance >3% in H for green taxa or >3% in P for red taxa) are in frames.

The clustering analysis (**Figure 3**) shows clearly separated branch of 10 patients with the most severe signs of the disease, nine of them (except for P11) being also selected by NMDS (**Figure 1**). The most distant branch of 16 probands, on the other hand, contains all 10 sH individuals and only one patient (P1) exhibiting only weak signs of the disease. The tree of the taxa revealed four major groups: 1) The small group of highly abundant taxa typical for periodontal health (genera *Streptococcus*, *Rothia*, *Veillonella*, and *Haemophilus parainfluenzae*) accompanied by a separate branch of a very special oral taxon—*Fusobacterium nucleatum*. The distribution of *F. nucleatum* differs from any other oral taxon because it is almost ubiquitous. It is difficult to find any oral sample completely lacking *F. nucleatum*. Nevertheless, in periodontal health, the relative abundance of fusobacteria is lower (usually not exceeding 10%) while in periodontitis it remarkably increases. 2) The second health-associated cluster comprises less abundant genera *Neisseria*, *Actinomyces*, *Kingella*, *Bergeyella*, *Lautropia*, *Streptococcus vestibularis*, and *Haemophilus paraphrohaemolyticus.* 3) The third cluster is clearly associated with the most diseased patients. It contains all red complex taxa together with several further strains of *Treponema* and genera *Fretibacterium*, *Filifactor*, and *Campylobacter*. 4) The last but most numerous cluster contains less abundant disease-associated taxa like *Mycoplasma*, *Dialister*, *Leptotrichia*, *Parvimonas*, *Desulfobulbus*, and others.

The heatmap clearly shows that in healthy individuals the disease-associated taxa are almost absent while in severe patients the health associated genera are replaced with a higher proportion of fusobacteria and selection of red taxa.

### The R/G Ratio Allows the Identification of Individuals in the Risk of Periodontitis Development

**Figure 4:**
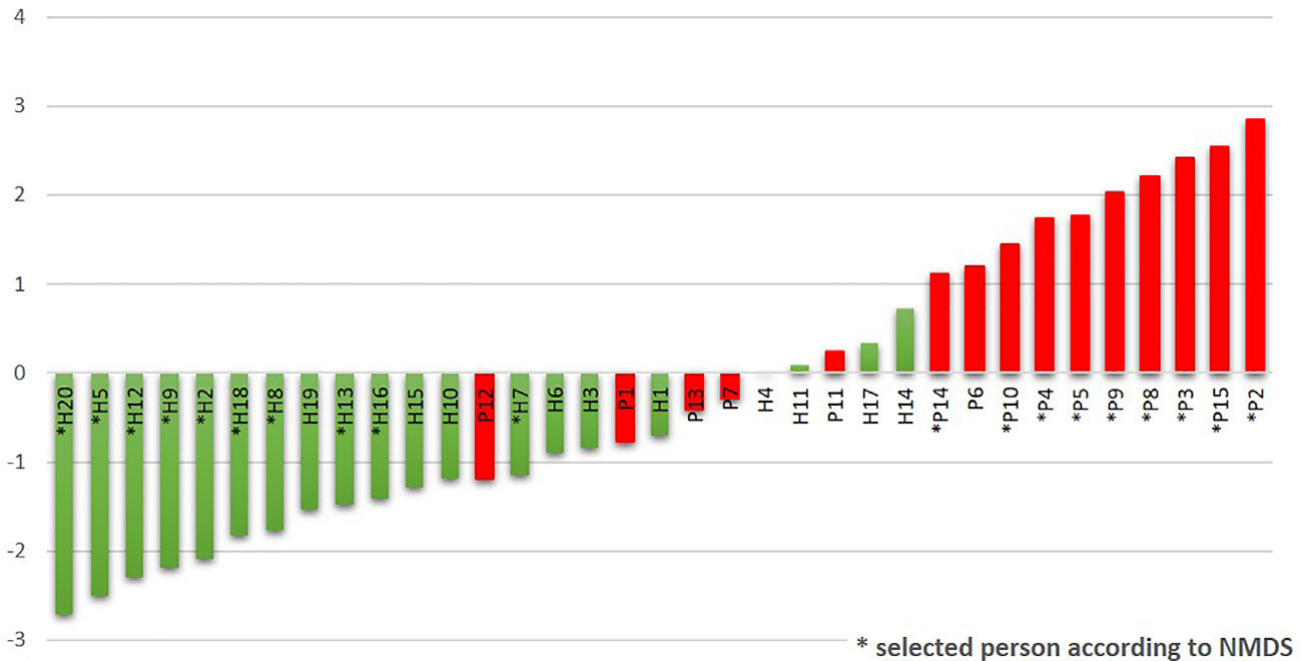
The R/G ratio calculated for the initial sampling (T0) results of the whole sets of tested subjects. The stars stand for samples also belonging to the narrowed selection set.

The R/G ratio was designed as a numerical expression of the health status. It was defined as the logarithm of a total relative abundance of red taxa (30R) divided by a total relative abundance of green taxa (17G, assigned according to **Figure 2B**), and it was calculated for each tested individual. The values were plotted in ascending order in green (for healthy individuals) and red (for patients) color (**Figure 4**). The periodontal health is clearly characterized by negative values while the periodontitis by positive ones, the highest numbers corresponding to the patients with the most severe signs of the disease. In the central region, both the patients and healthy individuals appear, with R/G ranging from −1 to +1. The plot not only describes the state of periodontal health for our full basic set of 20H and 15P individuals, but it could also simulate the chronology of changes in one individual OM on the way from periodontal health to severe periodontitis.

### The Predictive Ability of the R/G Value Was Confirmed on the Independent Set of 40 Samples

The testing set of 40 independent subgingival samples was obtained from 20 periodontally healthy individuals and 20 patients with periodontitis (having at least two periodontal pockets >5mm). The samples were marked TH1–20 and TP1–20. All samples were proceeded the same way as the above described full set of 20H + 15P and the R/G value was calculated for each sample to confirm the accuracy of the sample assignment to appropriate state of health (**Supplementary Table 5**). Complete OM profiles including R/G calculations are shown at lists “Testing set Healthy individuals” and “Testing set Periodontitis” of the “Table 2.xlsx” file, while the characteristics of each individual and R/G results are described at the list “Testing set all proband charact.” From 20 TH samples 9 exhibited the R/G value <−1, which we consider to reflect periodontal health and remaining 11 had the R/G value between −1 and +1, what could represent a transient state. The risk of periodontitis, however, is not considered to be high because none of samples exceeded the R/G value 0.25, rather the values were negative. From 20 TP samples 15 exhibited R/G value >+1 clearly indicating periodontal disease, three samples corresponded to the transient state but rather indicating the disease (the positive R/G value). Only two samples exhibited the R/G value typical for healthy individuals (−1.03 and −1.18), i.e. not corresponding to the diagnosed state of health. Summarized, 95% of samples were assigned correctly using the R/G value estimation.

### The Individual OM Varies in Time, But Generally Within Borders Given by the Health State

Before evaluating the OM variability in time we tested the ability of the used method to distinguish between the variability of results given by the methodology approach (sampling procedure, sample processing, and data analysis) and real natural dynamics of the OM composition. Two sets of initial T0 samples from healthy individuals (H1–H20; T0A and T0B) were processed independently and the results were compared to data obtained for identical sampling sites in later time points T1 and T2 (**Table 1**). The variability of OM profiles was expressed as the Bray-Curtis similarity index, and the R/G ratio was calculated for each sample individually. The Bray-Curtis indexes calculated for parallels of the first sampling (T0) ranged from 0.763 for H14 to 0.945 for H16, i.e., the parallels correspond well to each other, showing that the variable observations obtained for three sampling times correspond to the real temporal variability of the system and not the unreliability of the method. For further analysis, therefore, the sequencing results for T0A and T0B for each individual were merged and processed together.

**Table 1:** The individual dynamics of OM composition of healthy subjects in a horizon of 14 days (T1) and 2 months (T2).

Subject	Bray-Curtis index				R/G ratio		
	Replicate samples T0	T0-T1	T0-T2	T1-T2	T0	T1	T2
H1	0.813	0.657	0.644	0.548	–0.7	−0.99	−1.13
**H2**	**0.833**	**0.431**	**0.343**	**0.384**	−**2.08**	−**1.16**	−**1.99**
H3	0.902	0.291	0.27	0.232	−0.84	−0.08	0.55
H4	0.792	0.306	0.339	0.296	0.02	0.67	−0.78
**H5**	**0.915**	**0.599**	**0.34**	**0.317**	−**2.5**	−**2.39**	−**1.29**
H6	0.808	0.709	0.421	0.349	−0.9	−0.75	0.18
**H7**	**0.824**	**0.735**	**0.544**	**0.547**	−**1.14**	−**2.06**	−**1.46**
**H8**	**0.889**	**0.291**	**0.697**	**0.252**	−**1.77**	**0.2**	−**1.67**
**H9**	**0.897**	**0.682**	**0.729**	**0.544**	−**2.18**	−**1.38**	−**2.29**
H10	0.885	0.633	0.244	0.273	−1.19	−1.33	−2
H11	0.877	0.385	0.226	0.368	0.09	−1.04	−1.2
**H12**	**0.941**	**0.143**	**0.093**	**0.814**	−**2.3**	**−2.46**	**−2.66**
**H13**	**0.879**	**0.371**	**0.557**	**0.223**	**−1.48**	**−1.95**	**−1.57**
H14	0.763	0.195	0.183	0.458	0.72	−1.07	−1.18
H15	0.802	0.324	0.234	0.341	−1.28	−1.11	0.22
**H16**	**0.945**	**0.463**	**0.351**	**0.75**	**−1.41**	**−3.14**	**−1.51**
H17	0.845	0.402	0.555	0.389	0.34	0.38	0.68
**H18**	**0.859**	**0.339**	**0.71**	**0.44**	**−1.82**	**−0.64**	**−0.93**
H19	0.908	0.567	0.347	0.421	−1.53	−1.44	−0.31
**H20**	**0.935**	**0.578**	**0.537**	**0.624**	**−2.71**	**−2.32**	**−1.69**

Bray-Curtis index counted for parallels of the first sampling and for various samplings of each healthy individual. The numbers for subjects, which belong to sH sub-set are bolded. The R/G ratios are colored at the scale from green (healthy profile) to gray representing the transient state (values close to 0) and red (positive values up to four representing periodontitis profile).

The significantly lower values of Bray-Curtis index for different sampling times of each individual reflect the natural dynamics of OM composition, however, when considering also the R/G values (**Table 1**), it is evident, that the changes in the OM composition occur mainly within the range characterizing one state of periodontal health, i.e., for each clinically healthy individual the values correspond or to periodontal health (values <−1) or the transient state (values from −1 to +1). The results for 8 out of 10 healthy individuals belonging to sH group (bold in **Table 1**) show a stable level of R/G ratio within the range of periodontal health for all sampling times. A similar profile has been detected only for one additional healthy individual (H10). The dynamics of OM composition in periodontal health was visualized and compared to OM composition of periodontitis patients at **Supplementary Figure 1**. The microbiome in each oral cavity is not stable. It generally tends to change in a time span of weeks or months, nevertheless, it shows a tendency to keep within values typical for each state of health.

## Discussion

Since the high throughput sequencing methods were introduced to describe the oral microbiome, scientists have aimed to define the biomarker taxa with the potential to predict the risk of periodontitis development or to monitor the treatment efficiency. Even though none of the yet identified oral taxa occurs exclusively in one state of health, decades of research revealed taxa unambiguously assigned to periodontitis and opposite to periodontal health. The taxa significantly more abundant and prevalent in the diseased state comprise red complex bacteria *P. gingivalis*, *T. forsythia*, and *T. denticola* (Socransky et al., 1998), representatives of the genera *Fretibacterium* formerly marked as *Synergistetes*, *Peptostreptococcus*, *Desulfobulbus*, and further *Selenomonas sputigena* and *Filifactor alocis* (Abusleme et al., 2013; Kistler et al., 2013; Shi et al., 2015; Szafranski et al., 2015; Curtis et al., 2020). Genera *Actinomyces*, *Haemophilus*, or some representatives of *Neisseria*, *Rothia*, and *Streptococcus* were repeatedly assigned by the same authors to periodontal health. The taxa with the balanced average relative abundance and prevalence independent of the clinical state were by numerous authors called “core oral microbiome”. However, the list of core taxa differs from paper to paper starting with the poor two-membered core formed only by *Campylobacter gracilis* and *F. nucleatum ss. vincentii* (Curtis et al., 2020) to wider communities covering >10 genera including *Corynebacterium*, *Campylobacter*, *Lautropia*, *Veillonella*, *Rothia*, *Streptomyces*, *Neisseria*, *Leptotrichia*, *Treponema*, *Prevotella*, and others (Abusleme et al., 2013; Hong et al., 2015; Tsai et al., 2018).

**Figure 5:**
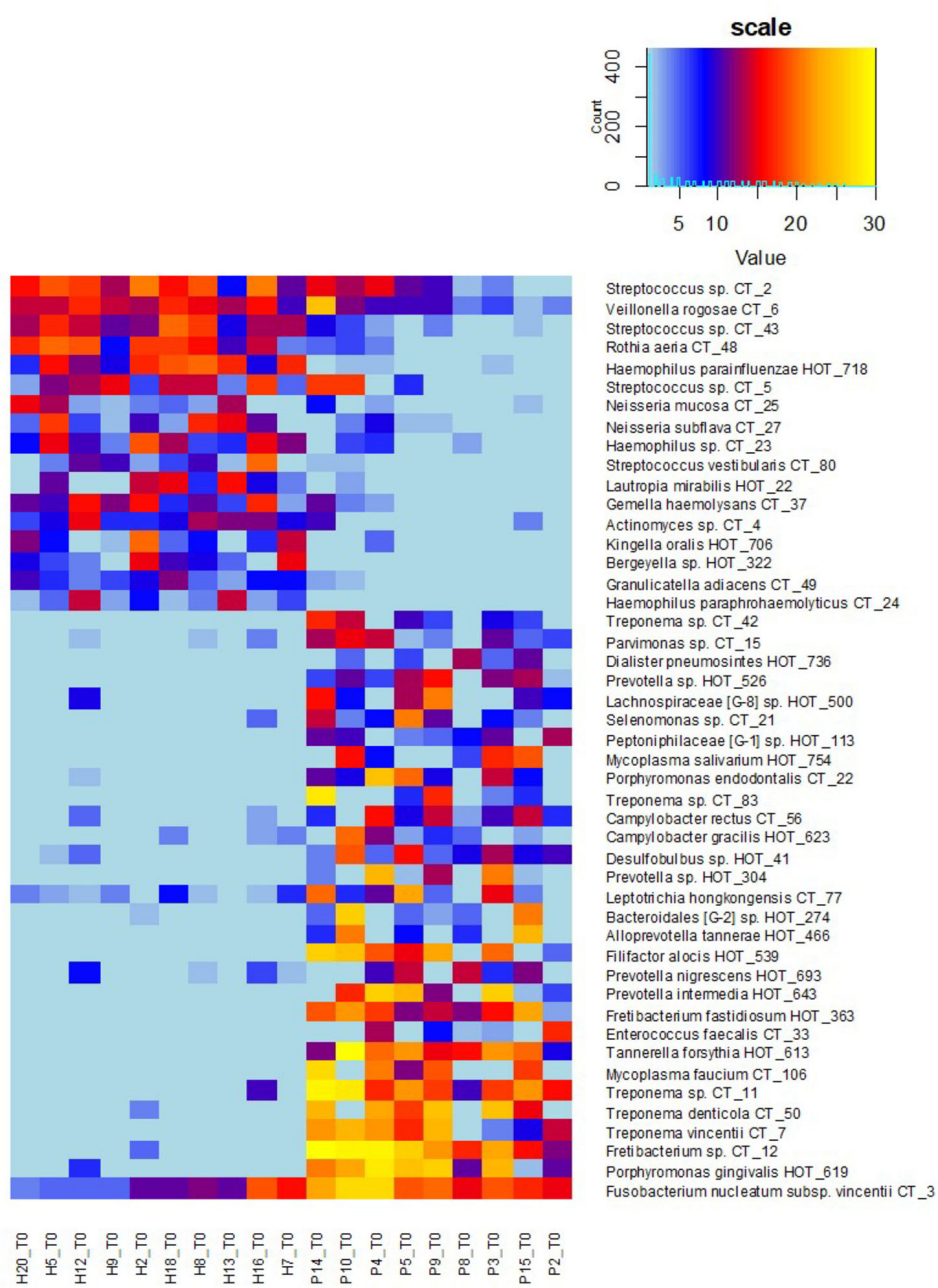
OM profiles of a narrowed sub-set of 10sH + 9sP at T0 based on R and G taxa relative abundance. The persons are ordered according to the increasing R/G ratio and the taxa according to the average relative abundance in H (green taxa) or P (red taxa) group.

Also in our case, when the full set of individuals (20H + 15P) was evaluated in time T0, the distribution of identified oral taxa resembled the model described by Abusleme (Abusleme et al., 2013) consisting of “green” (health-associated) taxa, “red” taxa associated with periodontitis, and “gray” core taxa exhibiting a balanced prevalence and average relative abundance across the whole set of tested individuals (**Figure 2A**). However, when we evaluated the narrowed sub-set of 9sP + 10sH persons with the most pronounced state of health, the gray “core” microbiome almost disappeared (**Figure 2B**) leaving only four low abundant taxa, i.e. the overlap of OMs of severe patients and truly healthy individuals is minimal (**Figure 5**).

We suppose that the extensive core OM described in the literature previously (Abusleme et al., 2013; Hong et al., 2015; Tsai et al., 2018) probably resulted from the inconsistency in the assignment of tested probands to periodontal health/periodontitis by individual authors and also logical enrolment of individuals without clinical signs of the disease but already having dysbiosis which could lead in future to periodontitis. The tested groups often differ in the minimal depth of periodontal pockets required for assignment to periodontitis (from >2 mm to >6 mm), and also the experience and individual approach of the examining periodontologist can play a role. Therefore it is difficult to distinguish whether the grey core taxa are rather ubiquitous independently of the medical condition or whether they represent the first signs of dysbiosis preceding the clinical manifestations of the disease or accompanying mild forms of periodontitis as suggested for example by Boutin et al. (2017).

To determine the R/G ratio as a numeric coefficient of the disease, we thus employed only 30 red (R) and 17 green (G) taxa defined on the selection sub-set. This approach should remarkably increase the probability of inclusion of only the taxa that are typical for both extreme conditions. The further estimation of the R/G ratio for the full set of tested individuals in T0 using this definition of R and G taxa resulted in a continuous graph with the most severe patients having R/G> +1 (often even 2–3) on one site and healthy individuals with R/G < −1 on the opposite side (**Figure 4**). The central part of the chart is occupied by individuals from both groups. It is evident that there exist not only the healthy and the diseased OMs but that the representation of R and G taxa can be arbitrarily variable and the R/G ratio can take on any value across the scale. We assume that this graph reflects the situation that could occur in one individual overtime during the gradual transition from health to chronic periodontitis. Unlike Abusleme et al. (2013) and in agreement with (Boutin et al., 2017) we thus suppose that the health associated (G) taxa are gradually overgrown/displaced by R taxa during the onset of the disease and that in severe patients the G taxa almost vanish. All H individuals have at least some R taxa, even though in very low relative abundance, as is clearly shown at the heatmap of selection sub-set samples (**Figure 5**), where the probands are ordered according to decreasing R/G. Under suitable conditions (for example poor oral hygiene, a shift in the immune system, smoking, change of the diet, aging-related changes) these R taxa can start to grow and shape local conditions enabling the growth of other R taxa and initiating dysbiosis which finally results in clinical signs of periodontitis (Boutin et al., 2017; Lamont et al., 2018). In the diagnosis, the overall complex view is thus essential and no currently commercially available method can be sufficient because they offer the identification of only a selected number of periodontal pathogens. Our findings are in agreement with the idea of PSD (polymicrobial synergy and dysbiosis) model (Hajishengallis and Lamont, 2012), showing that it is not sufficient to monitor one specific periopathogen or any selected group of pathogenic microorganisms. Instead, the OM should be considered a complex system which is, according to our findings dynamic in time, generally within values typical for the respective state of health but able to change directionally in case of starting dysbiosis. Repeated testing and R/G ratio estimation could thus serve as a diagnostic tool enabling early identification of individuals in the risk of periodontitis development. This was confirmed at the testing set of 40 independent samples obtained from 20 periodontally healthy individuals (TH1–20) and 20 patients with periodontitis (TP1–20). Only two samples, TP7 and TP9, were assigned incorrectly (**Supplementary Table 5**). A remarkable number of samples, however, exhibited values in the range from −1 to +1 which, according to our observations, indicates a higher probability of periodontitis development for clinically healthy individuals and possibly better prognosis for patients. In such cases, repeated testing and careful examination by experienced peridontologist could reveal the onset of the disease in time and help to decrease the danger of irreversible teeth loss.

The definition of biomarker taxa (R and G) is largely consistent with the findings of other authors (Camelo-Castillo et al., 2015; Szafranski et al., 2015; Meuric et al., 2017), however, any two publications mutually differ in the assignment of at least few individual taxa (Tsai et al., 2018). Szafranski assigned *Selenomonas noxia*, TM7_G1, and one *Fusobacterium* OTU to periodontal health while our results, in accordance with (Camelo-Castillo et al., 2015), indicate all these genera to be periodontitis associated, even though the relative abundance values of the TM7 and one *Selenomonas* taxon were low in our samples so that they did not fulfill the criteria of significance. Camelo-Castillo, on the other hand, assigns *Streptococcus sanguinis* to periodontal health and other streptococci such as *Streptococcus mitis* to the disease (Camelo-Castillo et al., 2015) while according to our results both *S. sanguinis* (CT43) and *S. mitis* (CT2) are associated with periodontal health. In individual cases, we identified CT2, CT5 (*S. parasanguinis*), and/or CT43 in the samples from periodontal pockets as well, but their prevalence and relative abundance conclusively prevail in periodontal health. In agreement with (Li et al., 2014; Camelo-Castillo et al., 2015) some members of the genus *Prevotella* were identified equally in H and P samples and two of them, *P. oris* and *P. histicola*, remained gray even when the selection sub-set was evaluated. Nevertheless, the majority of *Prevotella* species were in our study assigned to periodontitis including previously described members of orange complex *P. intermedia* and *P. nigrescens* (Socransky et al., 1998; Camelo-Castillo et al., 2015). Members of the genera *Actinomyces* and *Veillonella* were, on the other hand, assigned to periodontitis (Szafranski et al., 2015) while in our samples, similarly to, for example, Meuric et al. (2017), they clearly belong to periodontal health. The only exception was a low abundant *Actinomyces* sp. CT9 which prevalence among healthy samples was not convincing (one of the four gray taxa). Nevertheless, the role of anaerobic *Veillonella* sp. in the initiation of dysbiosis should be further investigated, because from **Figures 3** and **5** it is evident, that they frequently remain in the OM even when the typical health-associated taxa disappear and are replaced by highly abundant *Fusobacteria* and other periodontitis associated taxa.

Numerous authors tried to define a reliable measure of periodontal disease based on the taxonomic characterization of the OM (Shi et al., 2015; Szafranski et al., 2015; Meuric et al., 2017; Tsai et al., 2018). The mutual comparison of variable studies is not easy due to inconsistency in the estimation of periodontal health (varying from no periodontal pockets through the absence of periodontal pockets ≥3 mm to recovered sites primarily with periodontal pockets having the probing depth ≥6 mm), variable sampling and sample processing method, different 16S rDNA region to be sequenced and many other parameters. At the group level, all these tools clearly distinguished between periodontal health and periodontitis but, when individuals were considered, in 10–15% of cases (sometimes even 50%), the samples were misclassified or the classification was not possible due to insufficient strength of the method. Partially the misclassification could be caused by the above-mentioned inconsistency in the estimation of periodontal health and thus also by not optimal selection of marker taxa. Partially, it could be due to the individual unusual interplay between the oral microbiome and host immune system as for example in our sample H14 having typically pathogenic OM but no signs of the disease and on the other hand P13 with two periodontal pockets >6 mm but OM composition based on *Streptococci*, *Rothia*, and *Neisseria*. This was also the case of two misclassified testing samples TP7 and TP9.

Similar misclassification was observed by Szafranski et al. who diagnosed periodontitis using a jackknife method employing marker taxa defined by AUC or LEfSe method based on sequences of V1-2 or V5-6 region of 16S rDNA. Only the LEfSe with cut off 100 most abundant taxa provided statistically significant results and enabled reasonable assignment of the samples but still with four out of 19 samples (20%) excluded or misclassified. Also PCoA analysis led to misclassification of four samples, however, only two in agreement with the jackknife method. The other two clinically healthy women clustered with periodontitis patients, probably due to high relative abundance of *F. nucleatum* in one sample and even extreme relative abundance of *P. gingivalis* in the other (~25%). The reasonable comparison with our R/G method is impossible, because only three of nine evaluated patients with chronic periodontitis would meet also our criteria to be included in the periodontitis group and also the sample processing method differed remarkably.

Even more complicated would be the comparison with the method described by Shi et al. (2015), because they evaluate individual sites within the oral cavity independently, while all other cited authors (including this paper) consider the oral cavity rather a mutually interconnected system and consider the periodontitis status assignment valid for the individual as a whole. Moreover, the authors define their marker taxa based on the comparison of the affected sites with the recovered sites after SRP therapy and not to the healthy sites of periodontally healthy individuals. The discriminatory power of their classification approach was tested on both, the original set of diseased and recovered samples and on an independent set of fully processed samples (13 diseased + 1 recovered) and sequences of seven healthy individuals obtained from Human Microbiome Project. About 15% of samples were misclassified, what is comparable with the other studies, but additional 19 and 33% of samples of the original and independent set, respectively, were classified as uncertain. The authors suppose that this uncertainty results from a poor prediction strength, but considering our findings (continuous spectra of R/G values; **Figure 4**), it could rather reflect the transient state similarly to our R/G values from −1 to +1 at almost half of clinically healthy individuals.

The most similar approach to ours was applied to a big group of 422 healthy and 196 chronic periodontitis samples mined from papers and databases by Meuric et al. (2017). The authors used similar criteria for inclusion of individuals into groups (periodontal pockets ≤3mm = periodontal health; >5mm = periodontitis) to avoid possible overlap and ensure clear difference between both groups. Due to variability in the sequenced region in compared studies, the authors only could evaluate the results at the level of bacterial genera, what could bias the results in some cases (*Prevotella*, *Streptococcus*). Using clustering analysis and selection of marker genera, they assigned the original samples, as well as the control independent set of samples to the health state with ~85–90% accuracy (10% of periodontitis and 16% of health samples misclassified). The more frequent misclassification of healthy samples is not surprising because the dysbiosis precedes the clinical symptoms of the disease. Considering additionally a clear link of periodontitis to aging and very high frequency of the disease in developed countries, we can reasonably expect that in the group of periodontally healthy individuals with average age above ~45 years, a remarkable number of probands would already face the shift in the OM taxonomic composition towards anaerobic taxa associated with the disease.

The advantage of our approach is that for the first time, we do not rely only on clinical diagnosis, but based on the OM characterization and clustering analysis we select a narrowed set of the most diseased patients and healthy individuals with decreased probability of dysbiosis. And only this narrow set of individuals with the most pronounced state of health is employed to define the marker taxa. The proposed R/G index further enabled us to line up all tested individuals from the most affected to the “most healthy.” We believe that this continuous spectrum of taxonomic profiles mimics the temporal individual OM shift from truly healthy towards the dysbiotic and disease-associated OM, and it thus enables to reveal the individuals in risk of periodontitis development prior the first clinical symptoms. The method is highly robust and only remarkable shift in the OM taxonomic composition balance is reflected by a reasonable R/G value change.

The reliability of the R/G value based diagnosis would benefit in the future from an increased number of R and G taxa used for calculation, optimally defined on a wide set of well-defined samples originating from all over the world, because even if the testing set of individuals in this work originated from the same country as the full basic set (used for R and G definition), in several cases the identified R and G taxa represented only a small portion of the OM (less than 40%) what could make the diagnosis insecure. The ethnic and cultural differences could represent additional bias which should be evaluated before wider use of the method. Nevertheless, the observed 95% accuracy of the R/G value-based diagnosis seems to be highly acceptable. Moreover, it is reasonable to suppose that always there will remain a few specific cases (anatomic abnormalities or unusual OM-immune system interplay) that will resist the proper diagnosis based just on the OM evaluation independently of the size of well-defined R and G set of taxa.

## Data Availability Statement

The datasets for this study can be found in the NCBI Short Read Archive (BioProject accession no. PRJNA291567.

## Ethics Statement

The studies involving human participants were reviewed and approved by the Ethics Committee of the First Faculty of Medicine of Charles University and General University Hospital in Prague. The patients/participants provided their written informed consent to participate in this study.

## Author Contributions

JJ and JD postulated the hypothesis and designed the experiments, ZB and SP participated on experiment design, selection of probands, consulted and interpreted medical microbiology tasks, JM diagnosed the patients and performed sampling, TJ assisted in sampling and performed DNA isolation and primary PCR, GB and MK participated on primer design and selection and manuscript writing, LS prepared sequencing libraries for basic set of samples, together with JJ and LN analyzed results and formulated concept of R/G ratio. ML and BT processed samples for R/G method confirmation and evaluated the results, TV designed the pipeline for sequencing results analysis and participated on bioinformatics, LN performed bioinformatics analysis and together with LS wrote the manuscript. All authors critically read and commented on the manuscript. All authors contributed to the article and approved the submitted version.

## Funding

The study was supported by the Czech Health Research Council project 17-30753A (Ministry of Health, Czech Republic), grant 486417 from the Grant Agency of Charles University, by the project LQ1604 from National Sustainability Program II (project BIOCEV-FAR) and BIOCEV - CZ.1.05/1.1.00/02.0109.

## Conflict of Interest

The authors declare that the research was conducted in the absence of any commercial or financial relationships that could be construed as a potential conflict of interest.
