# Supplementary material for: R/G Value—A Numeric Index of Individual Periodontal Health and Oral Microbiome Dynamics
Source: Front Cell Infect Microbiol. 2021 Mar 10;11:602643. doi: 10.3389/fcimb.2021.602643 (PMC7988090; doi:10.3389/fcimb.2021.602643)
Supplement: Supplementary file 1 [file Image_1.pdf]

Supplementary Material

R/G value – a numeric index of individual periodontal health and oral microbiome dynamics

Najmanova Lucie[1†], Sabova Lenka[1†], Lenartova Magdalena[1,4], Janatova Tatjana[2], Mysak Jaroslav[2], Vetrovsky Tomas[1], Tesinska Barbora[1], Novotna Balikova Gabriela[3], Koberska Marketa[3], Broukal Zdenek[2], Duskova Jana[2], Podzimek Stepan[2], Janata Jiri[1,3*]

[1]Institute of Microbiology v. v. i., Czech Academy of Sciences, Videnska 1083, 142 20 Prague, Czech Republic

[2] Institute of Dental Medicine, First Faculty of Medicine, Charles University and General University Hospital in Prague, Karlovo namesti 32, Prague 2, Czech Republic

[3] Institute of Microbiology v. v. i., BIOCEV, Czech Academy of Sciences, Prumyslova 595, 252 50 Vestec , Czech Republic

[4]Department of Genetics and Microbiology, Faculty of Science, Charles University, Prague, Czech Republic

*** Correspondence:** Janata Jiri, janata@biomed.cas.cz

[†]These authors contributed equally to this work

1 Supplementary Figures

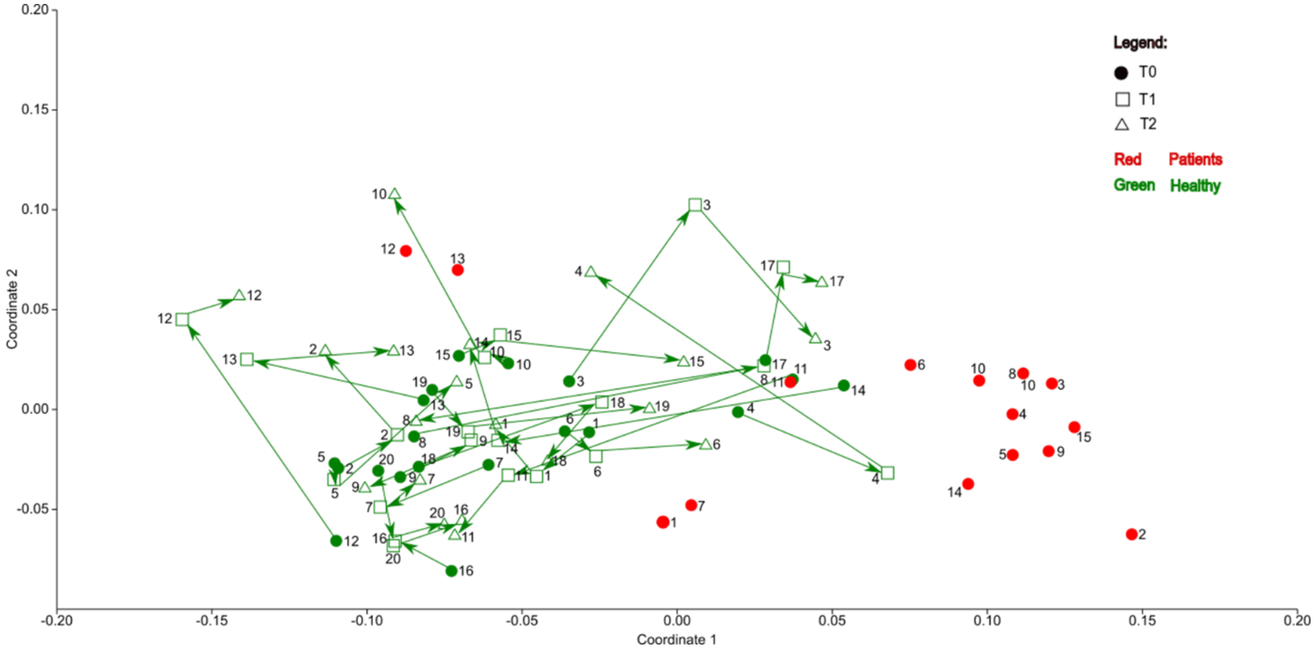

Supplementary Figure 1. Bray-Curtis distance based non-metric multidimensional scale analysis (NMDS) of full set of individual samples (20H + 15P) in all sampling times. The temporal samplings for each proband are connected with arrows.
