# Supplementary material for: R/G Value—A Numeric Index of Individual Periodontal Health and Oral Microbiome Dynamics
Source: Front Cell Infect Microbiol. 2021 Mar 10;11:602643. doi: 10.3389/fcimb.2021.602643 (PMC7988090; doi:10.3389/fcimb.2021.602643)
Supplement: Supplementary file 2 [file Table_1.docx]

Supplementary Material – Supplementary Table 1

R/G value – a numeric index of individual periodontal health and oral microbiome dynamics

Najmanova Lucie^1†^, Sabova Lenka^1†^, Lenartova Magdalena^1,4^, Janatova Tatjana^2^, Mysak Jaroslav^2^, Vetrovsky Tomas^1^, Tesinska Barbora^1^, Novotna Balikova Gabriela^3^, Koberska Marketa^3^, Broukal Zdenek^2^, Duskova Jana^2^, Podzimek Stepan^2^, Janata Jiri^1,3*^

^1^Institute of Microbiology v. v. i., Czech Academy of Sciences, Videnska 1083, 142 20 Prague, Czech Republic

^2^ Institute of Dental Medicine, First Faculty of Medicine, Charles University and General University Hospital in Prague, Karlovo namesti 32, Prague 2, Czech Republic

^3^ Institute of Microbiology v. v. i., BIOCEV, Czech Academy of Sciences, Prumyslova 595, 252 50 Vestec , Czech Republic

^4^Department of Genetics and Microbiology, Faculty of Science, Charles University, Prague, Czech Republic

*** Correspondence:** Janata Jiri, [janata@biomed.cas.cz](mailto:lucie.najmanova@biomed.cas.cz)

^†^These authors contributed equally to this work

**Supplementary Table 1. Group characteristics**

|  | **Group P (full set of patients with chronic periodontits)** | | | | | | | | |
| --- | --- | --- | --- | --- | --- | --- | --- | --- | --- |
| **Code** | **Age** | **Sex**  **F/M** | **No. of affected teeth/total no. of teeth** | **Sampled tooth/**  **PPD /mm/** | **localization of the periodontal pocket** | **BOP /%/** | | **CAL /mm/** | **CALCULATED**  **R/G (T0)** |
| P1 | 52 | F | 3/28 | 46/7 | M | 46 | | 8 | -0.77 |
| P2 | 48 | M | 20/20 | 46/8 | D | 60 | | 9 | **2.85** |
| P3 | 55 | M | 6/16 | 36/8 | M | 35 | | 10 | **2.42** |
| P4 | 46 | F | 6/26 | 46/7 | D | 86 | | 8 | **1.73** |
| P5 | 44 | M | 20/20 | 13/7 | M | 92 | | 10 | **1.77** |
| P6 | 45 | M | 4/28 | 47/9 | M | 20 | | 8 | 1.2 |
| P7 | 42 | F | 4/29 | 46/8 | M | 30 | | 10 | -0.28 |
| P8 | 52 | F | 4/27 | 27/9 | D | 80 | | 10 | **2.2** |
| P9 | 47 | F | 6/27 | 16/7 | MP | 96 | | 7 | **2.03** |
| P10 | 66 | F | 20/20 | 26/8 | M | 100 | | 10 | **1.45** |
| P11 | 45 | M | 2/22 | 12/9 | M | 54 | | 10 | 0.24 |
| P12 | 50 | M | 4/22 | 16/8 | M | 30 | | 10 | -1.18 |
| P13 | 44 | F | 2/28 | 16/7 | D | 48 | | 11 | -0.41 |
| P14 | 50 | M | 6/24 | 22/7 | M | 100 | | 8 | **1.11** |
| P15 | 49 | M | 6/32 | 17/8 | MP | 100 | | 10 | **2.54** |
|  | **Group H (full set of healthy individuals)** | | | | | | | | |
| **Code** | **Age** | **Sex**  **F/M** | **No. of affected teeth** | **Sampled tooth** |  | |  |  | **CALCULATED**  **R/G (T0)** |
| H1 | 44 | F | 0 | 13 |  | |  |  | -0.7 |
| H2 | 42 | F | 0 | 31 |  | |  |  | **-2.08** |
| H3 | 26 | F | 0 | 34 |  | |  |  | -0.84 |
| H4 | 31 | M | 0 | 46 |  | |  |  | 0.02 |
| H5 | 58 | M | 0 | 23 |  | |  |  | **-2.5** |
| H6 | 47 | M | 0 | 23 |  | |  |  | -0.9 |
| H7 | 33 | M | 0 | 33 |  | |  |  | **-1.14** |
| H8 | 44 | M | 0 | 45 |  | |  |  | **-1.77** |
| H9 | 40 | M | 0 | 35 |  | |  |  | **-2.18** |
| H10 | 38 | F | 0 | 15 |  | |  |  | -1.19 |
| H11 | 68 | M | 0 | 26 |  | |  |  | 0.09 |
| H12 | 30 | M | 0 | 13 |  | |  |  | **-2.3** |
| H13 | 31 | M | 0 | 22 |  | |  |  | **-1.48** |
| H14 | 44 | F | 0 | 45 |  | |  |  | 0.72 |
| H15 | 25 | M | 0 | 22 |  | |  |  | -1.28 |
| H16 | 28 | F | 0 | 13 |  | |  |  | **-1.41** |
| H17 | 33 | F | 0 | 44 |  | |  |  | 0.34 |
| H18 | 40 | F | 0 | 44 |  | |  |  | **-1.82** |
| H19 | 27 | F | 0 | 25 |  | |  |  | -1.53 |
| H20 | 37 | F | 0 | 13 |  | |  |  | **-2.71** |

**PPD = depth of the sampled periodonta pocket, BOP = bleeding on probing; CAL = clinical attachment loss;** No. of affected teeth = number of teeth with periodontal pockets >6 mm; Localization: M = mesial, D = distal, MP = mesio-palatial, Individuals selected by NMDS for a narrowed set of sH and sP are highlighted in grey.

During the experiment the state of periodontal health in the H group of individuals did not change.

The project design including the informed consent of the individuals was approved by the Ethics Committee of the General Faculty Hospital and First Medical Faculty, Charles University in Prague. The samples were anonymized and processed strictly under the assigned numbers.
