# Supplementary material for: R/G Value—A Numeric Index of Individual Periodontal Health and Oral Microbiome Dynamics
Source: Front Cell Infect Microbiol. 2021 Mar 10;11:602643. doi: 10.3389/fcimb.2021.602643 (PMC7988090; doi:10.3389/fcimb.2021.602643)
Supplement: Supplementary file 3 [file Table_2.docx]

Supplementary Material – Supplementary Table 2

R/G value – a numeric index of individual periodontal health and oral microbiome dynamics

Najmanova Lucie^1†^, Sabova Lenka^1†^, Lenartova Magdalena^1,4^, Janatova Tatjana^2^, Mysak Jaroslav^2^, Vetrovsky Tomas^1^, Tesinska Barbora^1^, Novotna Balikova Gabriela^3^, Koberska Marketa^3^, Broukal Zdenek^2^, Duskova Jana^2^, Podzimek Stepan^2^, Janata Jiri^1,3*^

^1^Institute of Microbiology v. v. i., Czech Academy of Sciences, Videnska 1083, 142 20 Prague, Czech Republic

^2^ Institute of Dental Medicine, First Faculty of Medicine, Charles University and General University Hospital in Prague, Karlovo namesti 32, Prague 2, Czech Republic

^3^ Institute of Microbiology v. v. i., BIOCEV, Czech Academy of Sciences, Prumyslova 595, 252 50 Vestec , Czech Republic

^4^Department of Genetics and Microbiology, Faculty of Science, Charles University, Prague, Czech Republic

*** Correspondence:** Janata Jiri, [janata@biomed.cas.cz](mailto:lucie.najmanova@biomed.cas.cz)

^†^These authors contributed equally to this work

**Supplementary Table 2.** **Primer sequences used in primary and secondary PCR**

| Primer | Sequence 5´- 3´ |
| --- | --- |
| **primary PCR** |  |
| eub530f | GTGCCAGCMGCNGCGG |
| eub1101r^*)^ | GGGGTTNCGNTCGTT |
| **secondary PCR** |  |
| T1eub530f | CCATCTCATCCCTGCGTGTCTCCGACTCAGACAGTGACTC**AGG**GTGCCAGCMGCNGCGG |
| T10eub530f | CCATCTCATCCCTGCGTGTCTCCGACTCAGTCTCTATGCG**AGG**GTGCCAGCMGCNGCGG |
| T11eub530f | CCATCTCATCCCTGCGTGTCTCCGACTCAGTGATACGTCT**AGG**GTGCCAGCMGCNGCGG |
| T13eub530f | CCATCTCATCCCTGCGTGTCTCCGACTCAGCATAGTAGTG**AGG**GTGCCAGCMGCNGCGG |
| T15eub530f | CCATCTCATCCCTGCGTGTCTCCGACTCAGATACGACGTA**AGG**GTGCCAGCMGCNGCGG |
| T19eub530f | CCATCTCATCCCTGCGTGTCTCCGACTCAGTGTACTACTC**AGG**GTGCCAGCMGCNGCGG |
| T20eub530f | CCATCTCATCCCTGCGTGTCTCCGACTCAGACGACTACAG**AGG**GTGCCAGCMGCNGCGG |
| T21eub530f | CCATCTCATCCCTGCGTGTCTCCGACTCAGCGTAGACTAG**AGG**GTGCCAGCMGCNGCGG |
| T22eub530f | CCATCTCATCCCTGCGTGTCTCCGACTCAGTACGAGTATG**AGG**GTGCCAGCMGCNGCGG |
| T23eub530f | CCATCTCATCCCTGCGTGTCTCCGACTCAGTACTCTCGTG**AGG**GTGCCAGCMGCNGCGG |
| T28eub530f | CCATCTCATCCCTGCGTGTCTCCGACTCAGACTACTATGT**AGG**GTGCCAGCMGCNGCGG |
| T33eub530f | CCATCTCATCCCTGCGTGTCTCCGACTCAGATAGAGTACT**AGG**GTGCCAGCMGCNGCGG |
| T36eub530f | CCATCTCATCCCTGCGTGTCTCCGACTCAGCGACGTGACT**AGG**GTGCCAGCMGCNGCGG |
| T37eub530f | CCATCTCATCCCTGCGTGTCTCCGACTCAGTACACACACT**AGG**GTGCCAGCMGCNGCGG |
| T39eub530f | CCATCTCATCCCTGCGTGTCTCCGACTCAGTACAGATCGT**AGG**GTGCCAGCMGCNGCGG |
| T41eub530f | CCATCTCATCCCTGCGTGTCTCCGACTCAGTAGTGTAGAT**AGG**GTGCCAGCMGCNGCGG |
| T44eub530f | CCATCTCATCCCTGCGTGTCTCCGACTCAGTCTAGCGACT**AGG**GTGCCAGCMGCNGCGG |
| T45eub530f | CCATCTCATCCCTGCGTGTCTCCGACTCAGTCTATACTAT**AGG**GTGCCAGCMGCNGCGG |
| T46eub530f | CCATCTCATCCCTGCGTGTCTCCGACTCAGTGACGTATGT**AGG**GTGCCAGCMGCNGCGG |
| T47eub530f | CCATCTCATCCCTGCGTGTCTCCGACTCAGTGTGAGTAGT**AGG**GTGCCAGCMGCNGCGG |
| T51eub530f | CCATCTCATCCCTGCGTGTCTCCGACTCAGAGCTCACGTA**AGG**GTGCCAGCMGCNGCGG |
| T52eub530f | CCATCTCATCCCTGCGTGTCTCCGACTCAGAGTATACATA**AGG**GTGCCAGCMGCNGCGG |
| T54eub530f | CCATCTCATCCCTGCGTGTCTCCGACTCAGAGTGCTACGA**AGG**GTGCCAGCMGCNGCGG |
| T55eub530f | CCATCTCATCCCTGCGTGTCTCCGACTCAGCGATCGTATA**AGG**GTGCCAGCMGCNGCGG |
| T57eub530f | CCATCTCATCCCTGCGTGTCTCCGACTCAGCGCGTATACA**AGG**GTGCCAGCMGCNGCGG |
| T58eub530f | CCATCTCATCCCTGCGTGTCTCCGACTCAGCGTACAGTCA**AGG**GTGCCAGCMGCNGCGG |
| T59eub530f | CCATCTCATCCCTGCGTGTCTCCGACTCAGCGTACTCAGA**AGG**GTGCCAGCMGCNGCGG |
| T62eub530f | CCATCTCATCCCTGCGTGTCTCCGACTCAGTACGTCATCA**AGG**GTGCCAGCMGCNGCGG |
| T64eub530f | CCATCTCATCCCTGCGTGTCTCCGACTCAGTATATATACA**AGG**GTGCCAGCMGCNGCGG |
| T65eub530f | CCATCTCATCCCTGCGTGTCTCCGACTCAGTATGCTAGTA**AGG**GTGCCAGCMGCNGCGG |
| T67eub530f | CCATCTCATCCCTGCGTGTCTCCGACTCAGTCGATAGTGA**AGG**GTGCCAGCMGCNGCGG |
| T70eub530f | CCATCTCATCCCTGCGTGTCTCCGACTCAGTGAGTCAGTA**AGG**GTGCCAGCMGCNGCGG |
| T71eub530f | CCATCTCATCCCTGCGTGTCTCCGACTCAGTGTAGTGTGA**AGG**GTGCCAGCMGCNGCGG |
| T77eub530f | CCATCTCATCCCTGCGTGTCTCCGACTCAGACGACAGCTC**AGG**GTGCCAGCMGCNGCGG |
| T79eub530f | CCATCTCATCCCTGCGTGTCTCCGACTCAGACTCATCTAC**AGG**GTGCCAGCMGCNGCGG |
| T81eub530f | CCATCTCATCCCTGCGTGTCTCCGACTCAGAGAGCGTCAC**AGG**GTGCCAGCMGCNGCGG |
| T83eub530f | CCATCTCATCCCTGCGTGTCTCCGACTCAGAGTAGTGATC**AGG**GTGCCAGCMGCNGCGG |
| T85eub530f | CCATCTCATCCCTGCGTGTCTCCGACTCAGAGTGTATGTC**AGG**GTGCCAGCMGCNGCGG |
| T98eub530f | CCATCTCATCCCTGCGTGTCTCCGACTCAGCTCTACGCTC**AGG**GTGCCAGCMGCNGCGG |
| T102eub530f | CCATCTCATCCCTGCGTGTCTCCGACTCAGTAGCTCTATC**AGG**GTGCCAGCMGCNGCGG |
| T107eub530f | CCATCTCATCCCTGCGTGTCTCCGACTCAGTCGAGCTCTC**AGG**GTGCCAGCMGCNGCGG |
| T115eub530f | CCATCTCATCCCTGCGTGTCTCCGACTCAGACTCACAGAG**AGG**GTGCCAGCMGCNGCGG |
| T117eub530f | CCATCTCATCCCTGCGTGTCTCCGACTCAGAGAGAGTGTG**AGG**GTGCCAGCMGCNGCGG |
| T124eub530f | CCATCTCATCCCTGCGTGTCTCCGACTCAGATCGTCTGTG**AGG**GTGCCAGCMGCNGCGG |
| T125eub530f | CCATCTCATCCCTGCGTGTCTCCGACTCAGATGTACGATG**AGG**GTGCCAGCMGCNGCGG |
| biotin-eub1101r | biotin-CCTATCCCCTGTGTGCCTTGGCAGTCTCAG**GTT**GGGGTTNCGNTCGTT |

Tag sequences are red, Adaptor A is blue, Adaptor B is purple; key to symbols: M = A or C, N = A or C or G or T; ^*)^ The original primer eub1100 (Dowd et al., 2008) was shifted one nucleotide downstream to overcome a frequent mismatch at the 3´end of the primer detected in silico for 16S rDNA sequences deposited in HOMD (for 21 percent of sequences).
