# Supplementary material for: R/G Value—A Numeric Index of Individual Periodontal Health and Oral Microbiome Dynamics
Source: Front Cell Infect Microbiol. 2021 Mar 10;11:602643. doi: 10.3389/fcimb.2021.602643 (PMC7988090; doi:10.3389/fcimb.2021.602643)
Supplement: Supplementary file 4 [file Table_3.docx]

Supplementary Material - Supplementary Table 3

R/G value – a numeric index of individual periodontal health and oral microbiome dynamics

Najmanova Lucie^1†^, Sabova Lenka^1†^, Lenartova Magdalena^1,4^, Janatova Tatjana^2^, Mysak Jaroslav^2^, Vetrovsky Tomas^1^, Tesinska Barbora^1^, Novotna Balikova Gabriela^3^, Koberska Marketa^3^, Broukal Zdenek^2^, Duskova Jana^2^, Podzimek Stepan^2^, Janata Jiri^1,3*^

^1^Institute of Microbiology v. v. i., Czech Academy of Sciences, Videnska 1083, 142 20 Prague, Czech Republic

^2^ Institute of Dental Medicine, First Faculty of Medicine, Charles University and General University Hospital in Prague, Karlovo namesti 32, Prague 2, Czech Republic

^3^ Institute of Microbiology v. v. i., BIOCEV, Czech Academy of Sciences, Prumyslova 595, 252 50 Vestec , Czech Republic

^4^Department of Genetics and Microbiology, Faculty of Science, Charles University, Prague, Czech Republic

*** Correspondence:** Janata Jiri, [janata@biomed.cas.cz](mailto:lucie.najmanova@biomed.cas.cz)

^†^These authors contributed equally to this work

**Supplementary Table 3. List of Combined Taxons with appropriate Oral Taxons**

| **Name of combined taxon (CT)** | **CT** | **Oral taxon (HOT)** | **Name of HOT** | **Clone/Strain** | **GenBank accession number** |
| --- | --- | --- | --- | --- | --- |
| Selenomonas noxia; S. infelix | 1 | HOT125 | Selenomonas flueggei |  |  |
|  |  | HOT126 | Selenomonas sp. |  |  |
|  |  | HOT130 | Selenomonas noxia |  |  |
|  |  | HOT138 | Selenomonas sp. |  |  |
|  |  | HOT139 | Selenomonas dianae |  |  |
|  |  | HOT146 | Selenomonas sp. |  |  |
|  |  | HOT479 | Selenomonas sp. |  |  |
|  |  | HOT481 | Selenomonas sp. |  |  |
|  |  | HOT639 | Selenomonas infelix |  |  |
|  |  | HOT726 | Centipeda periodontii |  |  |
|  |  | HOT892 | Selenomonas sp. |  |  |
|  |  | HOT919 | Selenomonas sp. |  |  |
|  |  | HOT920 | Selenomonas sp. |  |  |
|  |  | HOT936 | Selenomonas sp. |  |  |
|  |  | HOT937 | Selenomonas sp. |  |  |
| Streptococcus mitis; S. oralis | 2 | HOT058 | Streptococcus sp. |  |  |
|  |  | HOT061 | Streptococcus sp. |  |  |
|  |  | HOT064 | Streptococcus sp. |  |  |
|  |  | HOT070 | Streptococcus sp. |  |  |
|  |  | HOT071 | Streptococcus tigurinus |  |  |
|  |  | HOT074 | Streptococcus sp. |  |  |
|  |  | HOT398 | Streptococcus dentisani |  |  |
|  |  | HOT423 | Streptococcus sp. |  |  |
|  |  | HOT431 | Streptococcus sp. |  |  |
|  |  | HOT638 | Streptococcus infantis |  |  |
|  |  | HOT677 | Streptococcus mitis |  |  |
|  |  | HOT707 | Streptococcus oralis |  |  |
|  |  | HOT728 | Streptococcus peroris |  |  |
|  |  | HOT734 | Streptococcus pneumoniae |  |  |
|  |  | HOT948 | Streptococcus lactarius |  |  |
| Fusobacterium nucleatum | 3 | HOT200 | Fusobacterium nucleatum subsp. vincentii |  |  |
|  |  | HOT201 | Fusobacterium periodonticum |  |  |
|  |  | HOT202 | Fusobacterium nucleatum subsp. polymorphum |  |  |
|  |  | HOT203 | Fusobacterium sp. |  |  |
|  |  | HOT204 | Fusobacterium sp. |  |  |
|  |  | HOT205 | Fusobacterium sp. |  |  |
|  |  | HOT370 | Fusobacterium sp. |  |  |
|  |  | HOT420 | Fusobacterium nucleatum subsp. animalis |  |  |
|  |  | HOT689 | Fusobacterium naviforme |  |  |
|  |  | HOT698 | Fusobacterium nucleatum subsp. nucleatum |  |  |
| Actinomyces naeslundii | 4 | HOT169 | Actinomyces sp. |  |  |
|  |  | HOT170 | Actinomyces sp. |  |  |
|  |  | HOT171 | Actinomyces sp. |  |  |
|  |  | HOT175 | Actinomyces sp. |  |  |
|  |  | HOT176 | Actinomyces naeslundii |  |  |
|  |  | HOT688 | Actinomyces viscosus |  |  |
|  |  | HOT849 | Actinomyces johnsonii |  |  |
|  |  | HOT893 | Actinomyces oris |  |  |
| Streptococcus parasanguinis | 5 | HOT057 | Streptococcus sp. |  |  |
|  |  | HOT066 | Streptococcus sp. |  |  |
|  |  | HOT073 | Streptococcus australis |  |  |
|  |  | HOT411 | Streptococcus parasanguinis II |  |  |
|  |  | HOT578 | Streptococcus cristatus |  |  |
|  |  | HOT721 | Streptococcus parasanguinis I |  |  |
|  |  | HOT767 | Streptococcus sinensis |  |  |
|  |  | HOT886 | Streptococcus oligofermentans |  |  |
| Veillonella dispar; V. parvula | 6 | HOT158 | Veillonella rogosae |  |  |
|  |  | HOT160 | Veillonella dispar |  |  |
|  |  | HOT161 | Veillonella parvula |  |  |
|  |  | HOT524 | Veillonella atypica |  |  |
|  |  | HOT887 | Veillonella denticariosi |  |  |
| Treponema vincentii | 7 | HOT029 | Treponema vincentii |  |  |
|  |  | HOT226 | Treponema sp. |  |  |
|  |  | HOT228 | Treponema sp. |  |  |
|  |  | HOT230 | Treponema sp. |  |  |
|  |  | HOT231 | Treponema sp. |  |  |
|  |  | HOT236 | Treponema sp. |  |  |
|  |  | HOT237 | Treponema sp. |  |  |
|  |  | HOT667 | Treponema medium |  |  |
| Porphyromonas pasteri; P. catoniae | 8 | HOT275 | Porphyromonas sp. |  |  |
|  |  | HOT277 | Porphyromonas sp. |  |  |
|  |  | HOT278 | Porphyromonas sp. |  |  |
|  |  | HOT279 | Porphyromonas pasteri |  |  |
|  |  | HOT283 | Porphyromonas catoniae |  |  |
|  |  | HOT284 | Porphyromonas sp. |  |  |
| Actinomyces odontolyticus | 9 | HOT172 | Actinomyces sp. |  |  |
|  |  | HOT180 | Actinomyces sp. |  |  |
|  |  | HOT181 | Actinomyces lingnae [NVP] |  |  |
|  |  | HOT671 | Actinomyces meyeri |  |  |
|  |  | HOT701 | Actinomyces odontolyticus |  |  |
|  |  | HOT850 | Actinomyces cardiffensis |  |  |
| Prevotella histicola | 10 | HOT298 | Prevotella histicola |  |  |
|  |  | HOT306 | Prevotella sp. |  |  |
|  |  | HOT313 | Prevotella sp. |  |  |
|  |  | HOT314 | Prevotella sp. |  |  |
|  |  | HOT469 | Prevotella melaninogenica |  |  |
|  |  | HOT572 | Prevotella veroralis |  |  |
|  |  | HOT885 | Prevotella scopos |  |  |
| Treponema socranskii | 11 | HOT268 | Treponema sp. |  |  |
|  |  | HOT269 | Treponema sp. |  |  |
|  |  | HOT769 | Treponema socranskii |  |  |
| Fretibacterium sp. | 12 | HOT358 | Fretibacterium sp. |  |  |
|  |  | HOT359 | Fretibacterium sp. |  |  |
|  |  | HOT360 | Fretibacterium sp. |  |  |
|  |  | HOT361 | Fretibacterium sp. |  |  |
|  |  | HOT362 | Fretibacterium sp. |  |  |
| Aggregatibacter aphrophilus | 13 | HOT513 | Aggregatibacter sp. |  |  |
|  |  | HOT545 | Aggregatibacter aphrophilus |  |  |
|  |  | HOT720 | Aggregatibacter paraphrophilus |  |  |
|  |  | HOT898 | Aggregatibacter sp. |  |  |
|  |  | HOT946 | Haemophilus pittmaniae |  |  |
| Prevotella sp. | 14 | HOT292 | Prevotella sp. |  |  |
|  |  | HOT293 | Prevotella sp. |  |  |
|  |  | HOT300 | Prevotella sp. |  |  |
| Parvimonas micra | 15 | HOT110 | Parvimonas sp. |  |  |
|  |  | HOT111 | Parvimonas micra |  |  |
|  |  | HOT393 | Parvimonas sp. |  |  |
| Capnocytophaga sp. | 16 | HOT323 | Capnocytophaga sp. |  |  |
|  |  | HOT324 | Capnocytophaga sp. |  |  |
|  |  | HOT334 | Capnocytophaga sp. |  |  |
|  |  | HOT335 | Capnocytophaga sp. | Clone 069096_232 | GB JQ454775 |
|  |  | HOT336 | Capnocytophaga sp. | Strain F0502 | GB tbd |
|  |  | HOT412 | Capnocytophaga sp. | Clone C2MKM106 | GB AY278613 |
|  |  | HOT902 | Capnocytophaga sp. |  |  |
| Selenomonas sp. | 17 | HOT136 | Selenomonas sp. |  |  |
|  |  | HOT149 | Selenomonas sp. |  |  |
|  |  | HOT478 | Selenomonas sp. |  |  |
| Lactobacillus vaginalis; L. panis | 18 | HOT051 | Lactobacillus vaginalis |  |  |
|  |  | HOT709 | Lactobacillus oris |  |  |
|  |  | HOT818 | Lactobacillus reuteri genosp. 1 |  |  |
|  |  | HOT882 | Lactobacillus panis |  |  |
|  |  | HOT938 | Lactobacillus reuteri genosp. 2 |  |  |
| Lactobacillus brevis | 19 | HOT418 | Lactobacillus parafarraginis |  |  |
|  |  | HOT424 | Lactobacillus kisonensis |  |  |
|  |  | HOT558 | Lactobacillus brevis |  |  |
|  |  | HOT881 | Lactobacillus buchneri |  |  |
|  |  | HOT884 | Lactobacillus rapi |  |  |
| Selenomonas artemidis | 20 | HOT124 | Selenomonas artemidis |  |  |
|  |  | HOT133 | Selenomonas sp. |  |  |
|  |  | HOT137 | Selenomonas sp. |  |  |
| Selenomonas sputigena | 21 | HOT134 | Selenomonas sp. |  |  |
|  |  | HOT151 | Selenomonas sputigena |  |  |
|  |  | HOT442 | Selenomonas sp. |  |  |
| Porphyromonas endodontalis | 22 | HOT273 | Porphyromonas endodontalis |  |  |
|  |  | HOT285 | Porphyromonas sp. |  |  |
| Haemophilus haemolyticus | 23 | HOT036 | Haemophilus sp. |  |  |
|  |  | HOT851 | Haemophilus haemolyticus |  |  |
|  |  | HOT908 | Haemophilus sp. |  |  |
| Haemophilus sputorum | 24 | HOT035 | Haemophilus paraphrohaemolyticus |  |  |
|  |  | HOT944 | Haemophilus sputorum |  |  |
|  |  | HOT945 | Haemophilus parahaemolyticus |  |  |
| Neisseria flava; N. mucosa | 25 | HOT609 | Neisseria flava |  |  |
|  |  | HOT649 | Neisseria lactamica |  |  |
|  |  | HOT682 | Neisseria mucosa |  |  |
|  |  | HOT729 | Neisseria pharyngis |  |  |
|  |  | HOT764 | Neisseria sicca |  |  |
| Kingella denitrificans | 26 | HOT011 | Eikenella sp. |  |  |
|  |  | HOT012 | Kingella sp. |  |  |
|  |  | HOT582 | Kingella denitrificans |  |  |
| Neisseria subflava | 27 | HOT092 | Neisseria weaveri |  |  |
|  |  | HOT476 | Neisseria subflava |  |  |
|  |  | HOT610 | Neisseria flavescens |  |  |
|  |  | HOT669 | Neisseria meningitidis |  |  |
| Lachnoanaerobaculum orale | 28 | HOT082 | Lachnoanaerobaculum orale |  |  |
|  |  | HOT494 | Lachnoanaerobaculum saburreum |  |  |
| Capnocytophaga sp. | 29 | HOT336 | Capnocytophaga sp. | Clone X089 | GB AY005080 |
|  |  | HOT380 | Capnocytophaga sp. |  |  |
|  |  | HOT412 | Capnocytophaga sp. | Strain F0487 | GB tbd |
|  |  | HOT903 | Capnocytophaga sp. |  |  |
| Veillonellaceae [G-1] sp. | 30 | HOT129 | Veillonellaceae [G-1] sp. |  |  |
|  |  | HOT132 | Veillonellaceae [G-1] sp. |  |  |
|  |  | HOT150 | Veillonellaceae [G-1] sp. |  |  |
| Capnocytophaga sp. | 31 | HOT335 | Capnocytophaga sp. | Clone X066 | GB AY005078 |
|  |  | HOT336 | Capnocytophaga sp. | Clone 070035 613 | GB JQ462363 |
|  |  | HOT864 | Capnocytophaga sp. |  |  |
| Lactobacillus acidophilus; L. crispatus | 32 | HOT461 | Lactobacillus ultunensis |  |  |
|  |  | HOT529 | Lactobacillus acidophilus |  |  |
|  |  | HOT817 | Lactobacillus crispatus |  |  |
| Class Bacilli | 33 | HOT604 | Enterococcus faecalis |  |  |
|  |  | HOT801 | Enterococcus casseliflavus |  |  |
|  |  | HOT802 | Enterococcus saccharolyticus |  |  |
|  |  | HOT880 | Enterococcus durans |  |  |
| Leptotrichia wadei | 34 | HOT222 | Leptotrichia wadei |  |  |
|  |  | HOT417 | Leptotrichia sp. |  |  |
|  |  | HOT463 | Leptotrichia sp. |  |  |
| Treponema sp. | 35 | HOT250 | Treponema sp. |  |  |
|  |  | HOT251 | Treponema sp. |  |  |
|  |  | HOT517 | Treponema sp. |  |  |
|  |  | HOT951 | Treponema sp. |  |  |
| Veillonellaceae [G-1] sp. | 36 | HOT135 | Veillonellaceae [G-1] sp. | Clone ncd1699h10c1 | GB JF140932 |
|  |  | HOT145 | Veillonellaceae [G-1] sp. |  |  |
|  |  | HOT483 | Veillonellaceae [G-1] sp. |  |  |
|  |  | HOT918 | Veillonellaceae [G-1] sp. |  |  |
| Gemella morbillorum | 37 | HOT046 | Gemella morbillorum |  |  |
|  |  | HOT626 | Gemella haemolysans |  |  |
|  |  | HOT757 | Gemella sanguinis |  |  |
| Peptostreptococcaceae [XI][G-7] sp. | 38 | HOT106 | Peptostreptococcaceae [XI][G-7] [Eubacterium] yurii subsp. schtitka |  |  |
|  |  | HOT377 | Peptostreptococcaceae [XI][G-7] [Eubacterium] yurii |  |  |
|  |  | HOT922 | Peptostreptococcaceae [XI][G-7] sp. |  |  |
| Staphylococcus aureus; S. epidermidis | 39 | HOT076 | Staphylococcus warneri |  |  |
|  |  | HOT550 | Staphylococcus aureus |  |  |
|  |  | HOT567 | Staphylococcus caprae |  |  |
|  |  | HOT601 | Staphylococcus epidermidis |  |  |
| Veillonellaceae [G-1] sp. | 40 | HOT135 | Veillonellaceae [G-1] sp. | Clone DM071 | GB AF287790 |
|  |  | HOT148 | Veillonellaceae [G-1] sp. |  |  |
|  |  | HOT155 | Veillonellaceae [G-1] sp. |  |  |
| Klebsiella pneumoniae | 41 | HOT731 | Klebsiella pneumoniae |  |  |
|  |  | HOT865 | Kluyvera ascorbata |  |  |
| Treponema maltophilum | 42 | HOT260 | Treponema sp. |  |  |
|  |  | HOT664 | Treponema maltophilum |  |  |
| Streptococcus gordonii; S. sanguinis | 43 | HOT056 | Streptococcus sp. |  |  |
|  |  | HOT622 | Streptococcus gordonii |  |  |
|  |  | HOT758 | Streptococcus sanguinis |  |  |
| Peptococcus sp. | 44 | HOT167 | Peptococcus sp. |  |  |
|  |  | HOT168 | Peptococcus sp. |  |  |
| Actinomyces georgiae | 45 | HOT178 | Actinomyces sp. |  |  |
|  |  | HOT617 | Actinomyces georgiae |  |  |
|  |  | HOT877 | Actinomyces sp. |  |  |
| Mogibacterium timidum | 46 | HOT042 | Mogibacterium timidum |  |  |
|  |  | HOT593 | Mogibacterium diversum |  |  |
|  |  | HOT742 | Mogibacterium pumilum |  |  |
| Aggregatibacter segnis | 47 | HOT458 | Aggregatibacter sp. |  |  |
|  |  | HOT512 | Aggregatibacter sp. |  |  |
|  |  | HOT762 | Aggregatibacter segnis |  |  |
| Rothia dentocariosa | 48 | HOT188 | Rothia aeria |  |  |
|  |  | HOT587 | Rothia dentocariosa |  |  |
| Granulicatella adiacens | 49 | HOT534 | Granulicatella adiacens |  |  |
|  |  | HOT803 | Enterococcus italicus |  |  |
| Treponema denticola | 50 | HOT584 | Treponema denticola |  |  |
|  |  | HOT743 | Treponema putidum |  |  |
| Capnocytophaga granulosa | 51 | HOT325 | Capnocytophaga granulosa |  |  |
|  |  | HOT863 | Capnocytophaga sp. |  |  |
| Lactobacillus rhamnosus | 52 | HOT568 | Lactobacillus casei |  |  |
|  |  | HOT716 | Lactobacillus paracasei |  |  |
|  |  | HOT749 | Lactobacillus rhamnosus |  |  |
| Tannerella sp. | 53 | HOT286 | Tannerella sp. |  |  |
|  |  | HOT808 | Tannerella sp. |  |  |
|  |  | HOT916 | Tannerella sp. |  |  |
| Streptococcus anginosus | 54 | HOT543 | Streptococcus anginosus |  |  |
|  |  | HOT576 | Streptococcus constellatus |  |  |
|  |  | HOT644 | Streptococcus intermedius |  |  |
| Porphyromonas asaccharolytica | 55 | HOT547 | Porphyromonas asaccharolytica |  |  |
|  |  | HOT785 | Porphyromonas uenonis |  |  |
| Campylobacter rectus | 56 | HOT748 | Campylobacter rectus |  |  |
|  |  | HOT763 | Campylobacter showae |  |  |
| Alloprevotella sp. | 57 | HOT912 | Alloprevotella sp. |  |  |
|  |  | HOT913 | Alloprevotella sp. |  |  |
| Prevotella loescheii | 58 | HOT472 | Prevotella sp. |  |  |
|  |  | HOT658 | Prevotella loescheii |  |  |
| Haemophilus influenzae | 59 | HOT535 | Haemophilus aegyptius |  |  |
|  |  | HOT641 | Haemophilus influenzae |  |  |
| Campylobacter concisus | 60 | HOT575 | Campylobacter concisus |  |  |
|  |  | HOT580 | Campylobacter curvus |  |  |
| Peptostreptococcus stomatis | 61 | HOT112 | Peptostreptococcus stomatis |  |  |
|  |  | HOT542 | Peptostreptococcus anaerobius |  |  |
| Bifidobacterium dentium | 62 | HOT588 | Bifidobacterium dentium |  |  |
|  |  | HOT862 | Bifidobacterium longum |  |  |
|  |  | HOT889 | Bifidobacterium breve |  |  |
| Mycobacterium tuberculosis | 63 | HOT692 | Mycobacterium neoaurum |  |  |
|  |  | HOT822 | Mycobacterium tuberculosis |  |  |
|  |  | HOT823 | Mycobacterium leprae |  |  |
| Actinomyces gerencseriae | 64 | HOT618 | Actinomyces gerencseriae |  |  |
|  |  | HOT645 | Actinomyces israelii |  |  |
|  |  | HOT852 | Actinomyces massiliensis |  |  |
| Catonella morbi | 65 | HOT164 | Catonella sp. |  |  |
|  |  | HOT165 | Catonella morbi |  |  |
| Streptococcus sobrinus | 66 | HOT594 | Streptococcus downei |  |  |
|  |  | HOT768 | Streptococcus sobrinus |  |  |
| Neisseria gonorrhoeae | 67 | HOT621 | Neisseria gonorrhoeae |  |  |
|  |  | HOT737 | Neisseria polysaccharea |  |  |
| Pseudomonas aeruginosa | 68 | HOT536 | Pseudomonas aeruginosa |  |  |
|  |  | HOT834 | Pseudomonas otitidis |  |  |
| Desulfovibrio fairfieldensis | 69 | HOT040 | Desulfovibrio sp. |  |  |
|  |  | HOT605 | Desulfovibrio fairfieldensis |  |  |
| Fusobacterium necrophorum | 70 | HOT690 | Fusobacterium necrophorum |  |  |
|  |  | HOT860 | Fusobacterium gonidiaformans |  |  |
| Oribacterium parvum | 71 | HOT457 | Oribacterium sinus |  |  |
|  |  | HOT934 | Oribacterium parvum |  |  |
| Parascardovia denticolens | 72 | HOT586 | Parascardovia denticolens |  |  |
|  |  | HOT642 | Scardovia inopinata |  |  |
| Acinetobacter baumannii | 73 | HOT408 | Acinetobacter sp. |  |  |
|  |  | HOT554 | Acinetobacter baumannii |  |  |
| Propionibacterium acnes | 74 | HOT193 | Propionibacterium sp. |  |  |
|  |  | HOT530 | Propionibacterium acnes |  |  |
| Anaerococcus prevotii | 75 | HOT738 | Anaerococcus prevotii |  |  |
|  |  | HOT788 | Anaerococcus tetradius |  |  |
| Leptotrichia sp. | 76 | HOT212 | Leptotrichia sp. |  |  |
|  |  | HOT217 | Leptotrichia sp. |  |  |
| Leptotrichia hongkongensis | 77 | HOT213 | Leptotrichia hongkongensis |  |  |
|  |  | HOT879 | Leptotrichia sp. |  |  |
| TM7 [G-1] sp. | 78 | HOT346 | TM7 [G-1] sp. |  |  |
|  |  | HOT869 | TM7 [G-1] sp. |  |  |
| Achromobacter xylosoxidans | 79 | HOT343 | Achromobacter xylosoxidans |  |  |
|  |  | HOT828 | Bordetella pertussis |  |  |
| Streptococcus vestibularis; S. salivarius | 80 | HOT021 | Streptococcus vestibularis |  |  |
|  |  | HOT755 | Streptococcus salivarius |  |  |
| Treponema sp. | 81 | HOT235 | Treponema sp. |  |  |
|  |  | HOT242 | Treponema sp. |  |  |
| Alloprevotella sp. | 82 | HOT308 | Alloprevotella sp. |  |  |
|  |  | HOT914 | Alloprevotella sp. |  |  |
| Treponema sp. | 83 | HOT246 | Treponema sp. |  |  |
|  |  | HOT247 | Treponema sp. |  |  |
| Mogibacterium neglectum | 84 | HOT008 | Mogibacterium vescum |  |  |
|  |  | HOT691 | Mogibacterium neglectum |  |  |
| Erythromicrobium ramosum | 85 | HOT007 | Porphyrobacter tepidarius |  |  |
|  |  | HOT747 | Erythromicrobium ramosum |  |  |
| Bergeyella sp. | 86 | HOT900 | Bergeyella sp. |  |  |
|  |  | HOT907 | Bergeyella sp. |  |  |
| Enterobacter cancerogenus | 87 | HOT565 | Enterobacter cancerogenus |  |  |
|  |  | HOT634 | Enterobacter hormaechei |  |  |
| Lactobacillus johnsonii | 88 | HOT615 | Lactobacillus gasseri |  |  |
|  |  | HOT819 | Lactobacillus johnsonii |  |  |
| Mycoplasma pneumoniae | 89 | HOT616 | Mycoplasma genitalium |  |  |
|  |  | HOT732 | Mycoplasma pneumoniae |  |  |
| Lachnospiraceae [G-7] sp. | 90 | HOT086 | Lachnospiraceae [G-7] sp. |  |  |
|  |  | HOT163 | Lachnospiraceae [G-7] sp. |  |  |
| Treponema sp. | 91 | HOT263 | Treponema sp. |  |  |
|  |  | HOT264 | Treponema sp. |  |  |
| Gemella bergeri | 92 | HOT555 | Gemella bergeri |  |  |
|  |  | HOT928 | Gemella sp. |  |  |
| Peptostreptococcaceae [XI][G-4] sp. | 93 | HOT103 | Peptostreptococcaceae [XI][G-4] sp. |  |  |
|  |  | HOT369 | Peptostreptococcaceae [XI][G-4] sp. |  |  |
| Peptostreptococcaceae [XI][G-1] infirmum | 94 | HOT105 | Peptostreptococcaceae [XI][G-1] [Eubacterium] infirmum |  |  |
|  |  | HOT467 | Peptostreptococcaceae [XI][G-1] [Eubacterium] sulci |  |  |
| Corynebacterium tuscaniense | 95 | HOT184 | Corynebacterium tuscaniense |  |  |
|  |  | HOT835 | Corynebacterium mucifaciens |  |  |
| Microbacterium flavescens | 96 | HOT185 | Microbacterium ginsengisoli |  |  |
|  |  | HOT186 | Microbacterium flavescens |  |  |
| Stomatobaculum longum | 97 | HOT419 | Stomatobaculum longum |  |  |
|  |  | HOT910 | Stomatobaculum sp. |  |  |
| Propionibacterium propionicum | 98 | HOT194 | Propionibacterium sp. |  |  |
|  |  | HOT739 | Propionibacterium propionicum |  |  |
| TM7 [G-1] sp. | 99 | HOT352 | TM7 [G-1] sp. |  |  |
|  |  | HOT952 | TM7 [G-1] sp. |  |  |
| Pedobacter sp. | 100 | HOT321 | Pedobacter sp. |  |  |
|  |  | HOT933 | Pedobacter sp. |  |  |
| Leptotrichia hofstadii | 101 | HOT224 | Leptotrichia hofstadii |  |  |
|  |  | HOT909 | Leptotrichia sp. |  |  |
| Bifidobacterium subtile | 102 | HOT890 | Bifidobacterium subtile |  |  |
|  |  | HOT891 | Bifidobacterium scardovii |  |  |
| Ralstonia pickettii | 103 | HOT406 | Ralstonia sp. |  |  |
|  |  | HOT854 | Ralstonia pickettii |  |  |
| Lactobacillus plantarum | 104 | HOT861 | Lactobacillus plantarum |  |  |
|  |  | HOT883 | Lactobacillus pentosus |  |  |
| Neisseria sp. | 105 | HOT018 | Neisseria sp. |  |  |
|  |  | HOT499 | Neisseria sp. |  |  |
| Mycoplasma buccale | 106 | HOT561 | Mycoplasma buccale |  |  |
|  |  | HOT606 | Mycoplasma faucium |  |  |

Reference:

Dowd, S. E., Callaway, T. R., Wolcott, R. D., Sun, Y., McKeehan, T., Hagevoort, R. G., et al. (2008). Evaluation of the bacterial diversity in the feces of cattle using 16S rDNA bacterial tag-encoded FLX amplicon pyrosequencing (bTEFAP). *BMC Microbiol* 8, 125. doi:10.1186/1471-2180-8-125.
